# Altering Active-Site Loop Dynamics Enhances Standalone Activity of the Tryptophan Synthase Alpha Subunit

**DOI:** 10.1021/acscatal.4c04587

**Published:** 2024-11-02

**Authors:** Cristina Duran, Thomas Kinateder, Caroline Hiefinger, Reinhard Sterner, Sílvia Osuna

**Affiliations:** † Institut de Química Computacional i Catàlisi and Departament de Química, c/Maria Aurèlia Capmany 69, 17003 Girona, Spain; ‡ Institute of Biophysics and Physical Biochemistry, Regensburg Center for Biochemistry, University of Regensburg, D-93040 Regensburg, Germany; § ICREA, Pg. Lluís Companys 23, 08010 Barcelona, Spain

**Keywords:** tryptophan synthase, standalone activity, enzyme design, shortest path map (SPM) method, molecular dynamics (MD) simulations

## Abstract

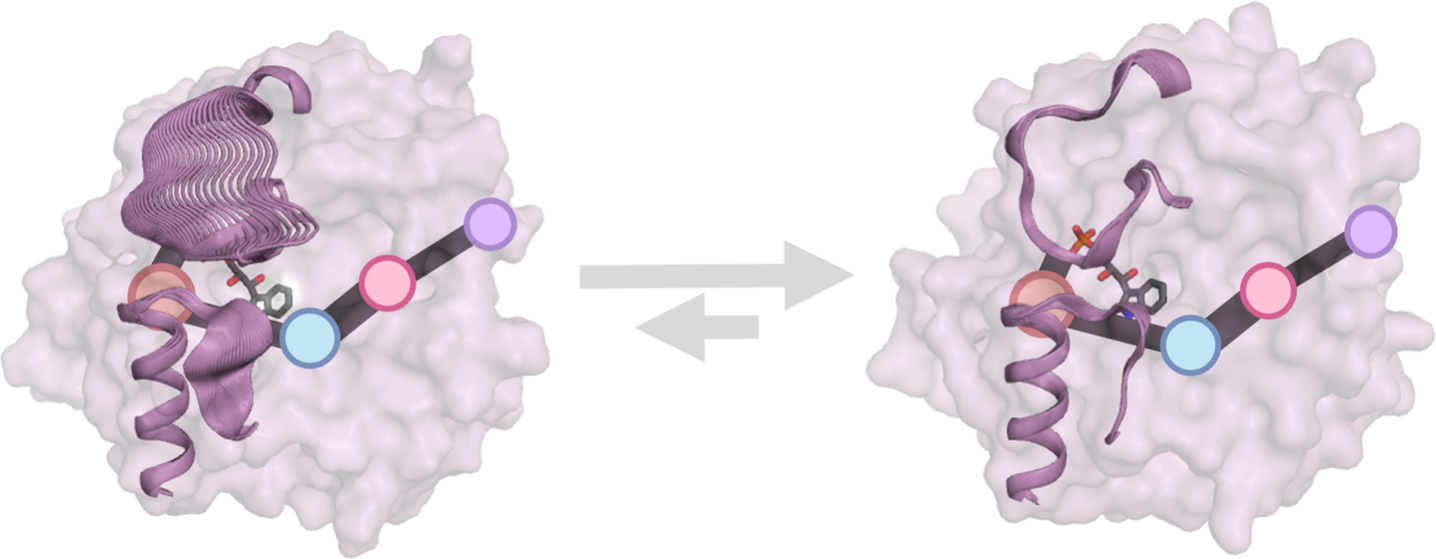

The α-subunit (TrpA) of the allosterically regulated bifunctional tryptophan synthase αββα enzyme catalyzes the retro-aldol cleavage of indole-glycerol phosphate (IGP) to d-glyceraldehyde 3-phosphate (G3P) and indole. The activity of the enzyme is highly dependent on the β-subunit (TrpB), which allosterically regulates and activates TrpA for enhanced function. This contrasts with the homologous BX1 enzyme from *Zea mays* that can catalyze the same reaction as TrpA without requiring the presence of any additional binding partner. In this study, we computationally evaluated and compared the conformational landscapes of the homologous *Zm*BX1 and *Zm*TrpA enzymes. Our results indicate that enhanced TrpA standalone activity requires the modulation of the conformational dynamics of two relevant active-site loops, loop 6 and 2, that need to be synchronized for accessing the catalytically activated closed state for IGP cleavage, as well as open states for favoring indole/G3P release. Taking as inspiration the evolutionary blueprint *Zm*BX1 and using our developed correlation-based tool shortest path map focused on the rate-determining conformational transition leading to the catalytically activated closed state, we computationally designed a variant named *Zm*TrpA^SPM4-L6BX1^, which displays a 163-fold improvement in catalytic efficiency for the retro-aldol cleavage of IGP. This study showcases the importance of fine-tuning the conformational dynamics of active-site loops for altering and improving function, especially in those cases in which a conformational change is rate determining.

## Introduction

Allostery is a central biological phenomenon, wherein two distinct sites within a biomolecule establish functional connections. This allosteric communication is particularly relevant in enzymatic mechanisms, where allosteric interactions frequently enhance processes such as enzyme–substrate binding and product release and exert a direct influence on catalytic turnover.^1−5^ Allosteric regulation alters the ensemble of conformations enzymes can adopt in solution, i.e., their conformational landscape is modified, which translates into a change in thermodynamic and dynamic properties.^6^ Some studies propose that allostery is an inherent characteristic of enzymes, as evidenced by the observation that mutations, which are distal from the enzyme active site, often lead to enhanced catalytic properties.^7^ Similar to the effect of allosteric regulation, these distal mutations induce a change in the enzyme conformational landscape, thus favoring the stabilization of key conformations for the novel activity.^8,9^

**Figure 1:**
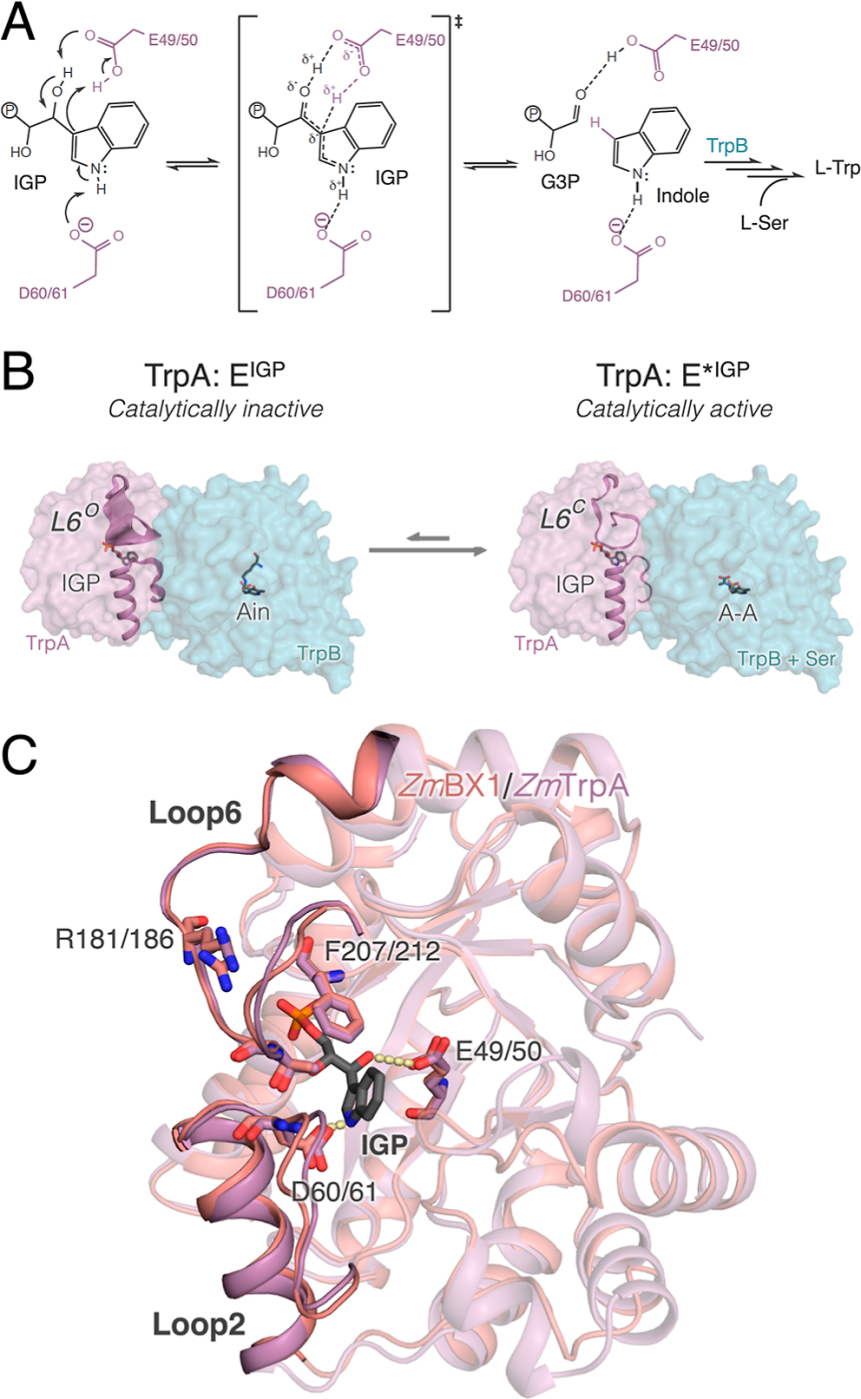
(A) Representation of the retro-adol reaction of IGP catalyzed by the α-subunit of tryptophan synthase (TrpA), yielding G3P and indole. Indole is then transferred through an internal tunnel to the active site of TrpB. (B) The activity of TrpA is allosterically regulated by TrpB. The open-to-closed (O-to-C) equilibrium of L6 in TrpA is shifted toward closed (L6^C^) conformations (i.e., the catalytically activated E*^IGP^ state is reached) when the amino acrylate intermediate (E(A–A)) is formed in TrpB in the presence of serine. The conformational change from E^IGP^(L6°) to E*^IGP^(L6^C^) was found to be rate determining for the TrpA reaction.^10,31^ It was postulated that the O-to-C equilibrium of TrpA is shifted toward open (L6°) catalytically unproductive states in the absence of TrpB or when the internal aldimine intermediate E(Ain) is formed in TrpB. IGP in TrpA and the reaction intermediate in TrpB are represented as sticks. (C) Overlay of *Zm*BX1 (in pink) and *Zm*TrpA (in purple) structures. Despite that they are structurally very similar, their standalone activity differs quite substantially. *Zm*BX1 is the evolutionary blueprint we take as inspiration for design. IGP, the catalytic Glu49/50 (*Zm*BX1/*Zm*TrpA numbering), Asp60/61, and key residues Arg181/186 and Phe207/212 are represented as sticks.

Allosteric regulation within multimeric enzyme complexes renders the isolated subunits highly inefficient; i.e., their standalone activity is extremely poor. This observation is exemplified in the heterodimeric enzyme complex tryptophan synthase (TrpS), which is composed of two α- and two β-subunits (TrpA and TrpB) arranged in an αββα configuration. TrpA catalyzes the retro-aldol cleavage of indole glycerol phosphate (IGP), yielding d-glyceraldehyde 3-phosphate (G3P) and indole (Figure 1A). The reversible cleavage of IGP is believed to proceed via “push–pull” general acid–base catalysis involving the residues Asp61 and Glu50.^10,11^ Then, an internal TrpA–TrpB tunnel is used by indole to ultimately reach the TrpB subunit containing the pyridoxal-5′-phosphate (PLP) cofactor that assists its condensation with l-serine for l-tryptophan formation. The existing tight allosteric TrpA–TrpB regulation involves the shift between open (catalytically nonproductive) and closed (catalytically productive) conformational states of the active sites, whose equilibrium depends on the ligand present in TrpA and the covalently bound intermediate in TrpB (Figure 1B).^12−18^

The design of standalone TrpBs revealed that distal mutations were needed to recover the open-to-closed conformational ensemble of the TrpB enzyme found in complex with TrpA.^16,19,20^ Interestingly, the reconstruction of a TrpS phylogenetic tree revealed that ancestral TrpB variants were inactivated in the presence of TrpA.^21,22^ This allosteric inactivation progressively turned into activation along the evolutionary trajectory. Multiple Sequence Alignments (MSAs) applied on the reconstructed phylogenetic tree allowed the identification of a subset of positions not close to the active site but which are important for switching the allosteric regulation.^21^ The key role of remote mutations in fine-tuning TrpB standalone activity prompted us to apply our developed correlation-based tool Shortest Path Map (SPM).^8,23^ SPM was used in previous studies for identifying key conformationally relevant positions (either at the active site or at distal sites) important for allostery,^24−26^ but also in combination with MSA for designing standalone TrpB variants and highly efficient esterases from hydroxynitrile lyases.^27,28^

The change in allosteric regulation along the phylogenetic tree identified for TrpB is, however, not observed in the case of TrpA.^21^ The Last Bacterial Common Ancestor TrpA is already allosterically activated by TrpB, which makes the identification of key positions for standalone activity via an MSA much more challenging.^21^ The low activity of TrpA in absence of the TrpB binding partner was hypothesized to be related to the inability of TrpA to adopt the productively closed conformation of the active site in absence of TrpB, which is mostly accomplished by loop 6 (L6), and to some extent loop 2 (L2) that contains the catalytic residue Asp61 (Figure 1B).^10,29^ The open-to-closed equilibrium of TrpA is shifted toward closed conformations when the aminoacrylate intermediate (E(A–A)) is formed in TrpB (Figure 1B).^10,30^ The formation of E(A–A) at TrpB promotes a conformational change in TrpA that enhances the rate of IGP cleavage 150-fold.^31^ The study of the kinetics of TrpA in the presence of serine and TrpB revealed that the retro-aldol cleavage is not rate determining, but instead it is the transition from the catalytically inactive (E^IGP^) to the activated conformation (E*^IGP^).^10,31^ This conformational change was hypothesized to be the open-to-closed transition of L6.^10^ These findings suggest that the closed conformation of especially L6 should be stabilized for generating TrpA variants with enhanced standalone activity, which are less dependent on the activation by TrpB and the reaction intermediate bound to TrpB.

Nature already presents a standalone enzyme exhibiting high activity for the retro-aldol cleavage of IGP in the absence of any additional interaction partner. The TrpA paralogue from maize, *Zea mays* BX1, is structurally very similar to TrpA (Figure 1C) and shares a sequence identity with *Zm*TrpA of 63.3%. In fact, both enzymes contain identical catalytic residues (i.e., Glu49/Glu50 and Asp60/Asp61 for *Zm*BX1/*Zm*TrpA).^11^ Available X-ray structures of *Zm*BX1 present L6 either in an open or a closed conformation, thus suggesting that *Zm*BX1 in the absence of any additional binding partner can adopt the essential closed catalytically activated E*^IGP^ conformation for catalysis.^10^ To confirm the key role of *Zm*BX1 L6 for standalone activity, we implanted L6 from *Zm*BX1 into *Zm*TrpA, resulting in the variant *Zm*TrpA_L6zmBX1,^32^ which was renamed here to *Zm*TrpA^L6BX1^. This resulted in a strong increase of the catalytic constant *k*_cat_ but a rather high *K*_M_, thus providing a *k*_cat_/*K*_M_ that did remain far behind the catalytic efficiencies of *Zm*BX1 and *Zm*TrpA in complex with *Zm*TrpB, thus indicating that additional mutations are needed to reach such high levels of activity.

In this study, we rationalize the role played by L6 and L2 in *Zm*BX1 for conferring enhanced standalone activity. Using this information and taking the evolutionary blueprint *Zm*BX1 as inspiration, we then rationally design a new TrpA variant starting from the previously generated *Zm*TrpA^L6BX1^ variant.^32^ As the rate-determining step in TrpA is the formation of the catalytically activated E*^IGP^ state,^10,31^ we first computationally reconstruct the closed-to-open conformational landscape of L6 and L2 of *Zm*BX1, *Zm*TrpA, and *Zm*TrpA^L6BX1^ in the absence and presence of IGP. We identify conformationally relevant positions via SPM calculation considering the catalytically activated closed state and compare how the networks of intramolecular pathways differ between the *Zm*TrpA^L6BX1^ starting scaffold and the reference *Zm*BX1. A carefully selected subset of conformationally relevant, nonconserved SPM positions between both enzymes is then used for designing the *Zm*TrpA^SPM4-L6BX1^ variant that shows a further enhanced catalytic efficiency for IGP cleavage. This work demonstrates the potential of our SPM methodology to identify conformationally relevant active site and distal positions for enhanced standalone activity. Although we focused on TrpA engineering, we anticipate that the developed methodology can be generally applied, being particularly relevant for those enzyme cases in which a conformational change is rate determining.

## Results and Discussion

### *Zm*BX1 Adopts Catalytically Productive Closed Conformations

**Figure 2:**
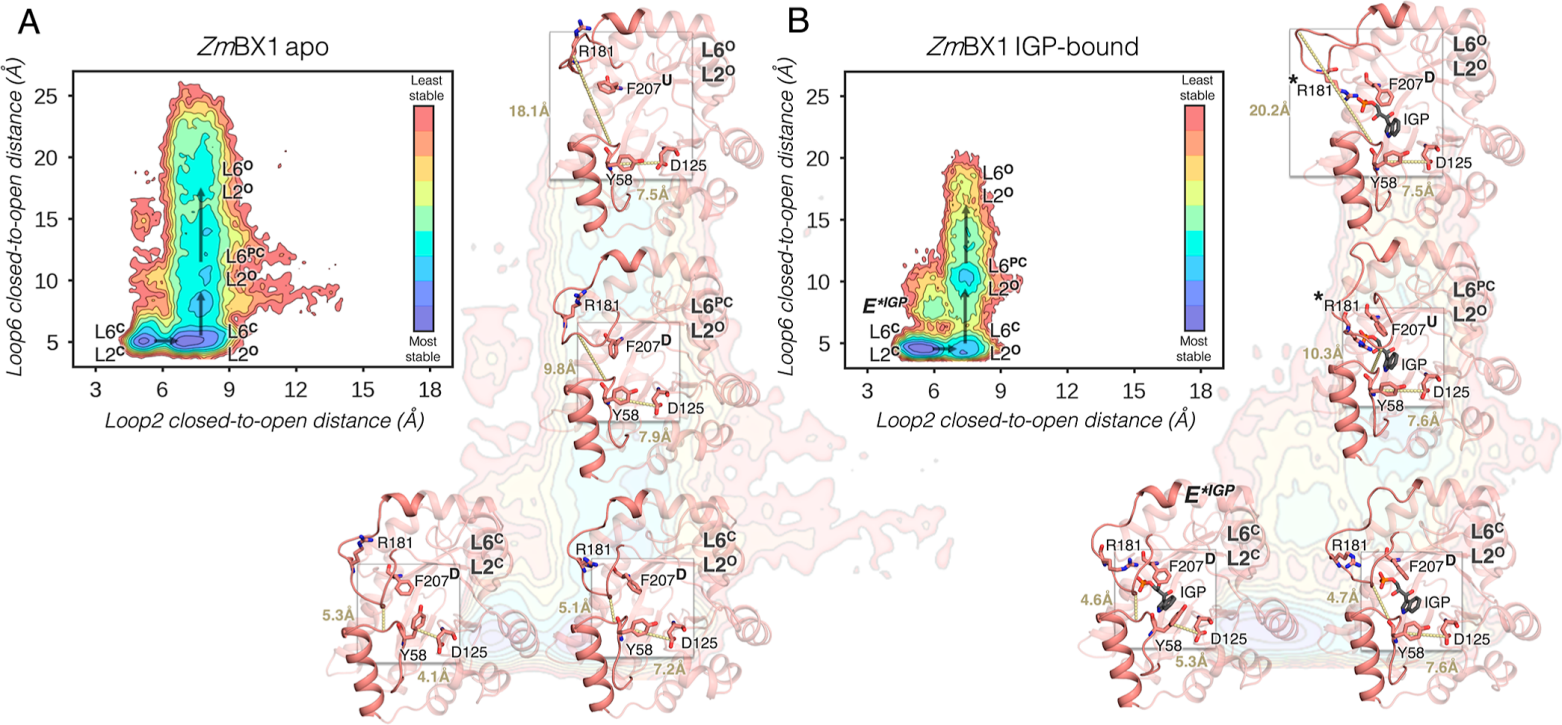
Reconstructed FEL of *Zm*BX1 in the (A) absence and (B) presence of the substrate IGP. For FEL reconstruction, the distance between Thr178 and Gly61 residues, that describes the closed-to-open transition of L6 (*y* axis), and the distance between Tyr58 and Asp125 for L2 opening (*x* axis) are used. Most stable conformations are colored in blue, whereas the least stable ones are depicted in red. Each minimum in the FEL is labeled according to the open (O)/closed (C) conformation of L6 and L2. The catalytically activated E*^IGP^ presenting both L6 and L2 in a closed conformation is labeled as E*^IGP^**(L6**^**C**^**L2**^**C**^). A representative structure extracted from each labeled minimum from the FEL reconstructed via multiple replica MD simulations (10 replicas of 500 ns) is shown and the average distance for the two L6 and L2 closed-to-open distances (*y*, *x* axis in panel A and B) is included. The following residues are represented in sticks: Tyr58 and Asp125 for L2, and the key residues for substrate binding/product release Arg181 and Phe207. The different conformations of Phe207 are marked with up (U)/down (D) to easily identify the differences in their side chain conformation. In panel (B), IGP is also represented as sticks. Those conformations in which a salt bridge is established between the phosphate group of IGP and Arg181 are marked with a star (*).

Intrigued by how *Zm*BX1 achieves the high level of catalytic activity for IGP cleavage in the absence of any additional binding partner, we decided to reconstruct the closed-to-open conformational landscape of L6 and L2 via nanosecond time scale Molecular Dynamics (MD) simulations. We performed 10 replicas of 500 ns in the apo- and IGP-bound states for *Zm*BX1 and all variants (see the Supporting Information). To this end, we selected several key variables that describe the conformations of L6 and L2 and the reorganization of the active-site pocket: the distance between Thr178 and Gly61 residues, that describes the closed-to-open transition of L6 (*y* axis in Figure 2), and the distance between Tyr58 and Asp125, that describes the closed-to-open transition of L2 (*x* axis). Our computed free energy landscape (FEL) starting from the closed conformation of L6 and L2 shows that in the absence of any ligand, *Zm*BX1 can adopt not only the catalytically productive closed conformation of L6 and L2 E*(**L6**^**C**^**L2**^**C**^) but also an additional conformation in which L6 is closed and L2 is open (**L6**^**C**^**L2**^**O**^) (Figure 2A). Starting from this open conformation of L2, the opening of L6 is facilitated, and in fact, multiple conformations presenting long L6 distances are visited. Open conformations of L6 and L2 might be important for IGP binding and G3P/indole release. Interestingly, the opening of L6 induces a reorganization of Phe207 and Arg181. As shown in Figure 2A, in closed states of L6, Phe207 adopts a conformation in which the side chain is in the active site pocket (*down* conformation). However, the opening of L6 favors the positioning of Phe207 pointing away from the active site in an *up* conformation, which provides additional space for substrate binding. By careful inspection of the available X-ray structures of *Zm*BX1, we realized that indeed the two open and closed conformations of L6 induce a change in the side chain conformation of Phe207 located in the active site. This is also observed in the available structures of TrpAs reported (Figure S1). This rearrangement also affects the conformation of Arg181, which gets solvent exposed when L6 is open. We hypothesize that this new conformation of Arg181 and Phe207 after the L6 and L2 opening might be important for substrate binding and product release. In our reconstructed FEL, open states of L6 and L2 are also visited although they are substantially less stable than the closed states of L6 (i.e., E*(L6^**C**^**L2**^**C**^**)** and **L6**^**C**^**L2**^**O**^). We additionally ran MD simulations starting from the other reported crystal structure, which features a **L6**^**O**^**L2**^**O**^ state (Figure S2). These simulations indicate that the **L6**^**O**^**L2**^**O**^ state is rather stable and in fact the transition from the **L6**^**O**^**L2**^**O**^ state to the **L6**^**C**^**L2**^**C**^ state is not observed, thus suggesting a higher stability for open states of L6 and L2. These findings are in line with the hypothesized ensemble of open and closed states of TrpA, according to which the open, catalytically unproductive state is the most stable one.^10^ Altogether, this analysis indicates that *Zm*BX1 in the absence of any ligand and additional binding partner can effectively sample not only the closed conformation of L6 and L2, which is important for IGP cleavage, but also the open states of L2 and L6, which are crucial for substrate binding and product release.

To investigate how the conformational landscape of *Zm*BX1 is altered in the presence of IGP and how it can efficiently catalyze the retro-aldol cleavage of IGP, we performed MD simulations with the catalytically activated E*^IGP^ state, i.e., IGP bound in the **L6**^**C**^**L2**^**C**^ conformation (E*^IGP^**(L6**^**C**^**L2**^**C**^**)**, Figure 2B). The most populated minima in the IGP-bound state contain both L6 and L2 closed (E*^IGP^**(L6**^**C**^**L2**^**C**^**)** in FEL). In this state, the catalytic distance between IGP and Glu49, which is suggested to have a dual role as a proton donor and acceptor, is 4.0 ± 0.4 Å. L2 contains the catalytically relevant Asp60, which is suggested to abstract the hydrogen of N1 of the indole ring for indolenine tautomerization (Figure 1A), and thus, the closed state of L2 is also important for catalysis. The distance between N1 of the indole ring of IGP and Asp60 in the E*^IGP^**(L6**^**C**^**L2**^**C**^**)** state is 3.8 ± 0.3 Å (Figure S3). However, as observed in the apo state, L2 can easily transition to more open conformations that might be important for IGP binding and G3P release (**L6**^**C**^**L2**^**O**^ and **L6**^**O**^**L2**^**O**^ states). The closed-to-open transition of L6 from the **L6**^**C**^**L2**^**O**^ state is easier than that from the fully closed conformation (E*^IGP^**(L6**^**C**^**L2**^**C**^**)**). This closed-to-open conformational change of L6 involves a change of conformation of Arg181, which establishes a salt bridge with the phosphate group of IGP, thus potentially contributing to IGP binding and G3P release. Interestingly, it was found that the position of this Arg181 within the sequence of L6 was different in *Zm*BX1 compared to *St*TrpA from *Salmonella typhimurium* and related TrpAs (*Zm*BX1 and *Zm*TrpA contain Arg181 in the same location).^10^ Based on this observation, it was hypothesized that the position of Arg181 was essential for achieving faster kinetics for TrpA.^10^ Indeed, our simulations suggest that Arg181 can play a crucial role in assisting the binding and release of the phosphate-containing ligands (IGP and G3P). As observed for the apo state, the change in conformation of Arg181 also involves positioning Phe212 in an *up* conformation, as described above.

### Transfer of BX1 L6 Enhances Standalone Catalytic Activity of *Zm*TrpA by Synchronizing L2/L6 Dynamics and Stabilizing the Catalytically Activated E*^IGP^ State

**Figure 3:**
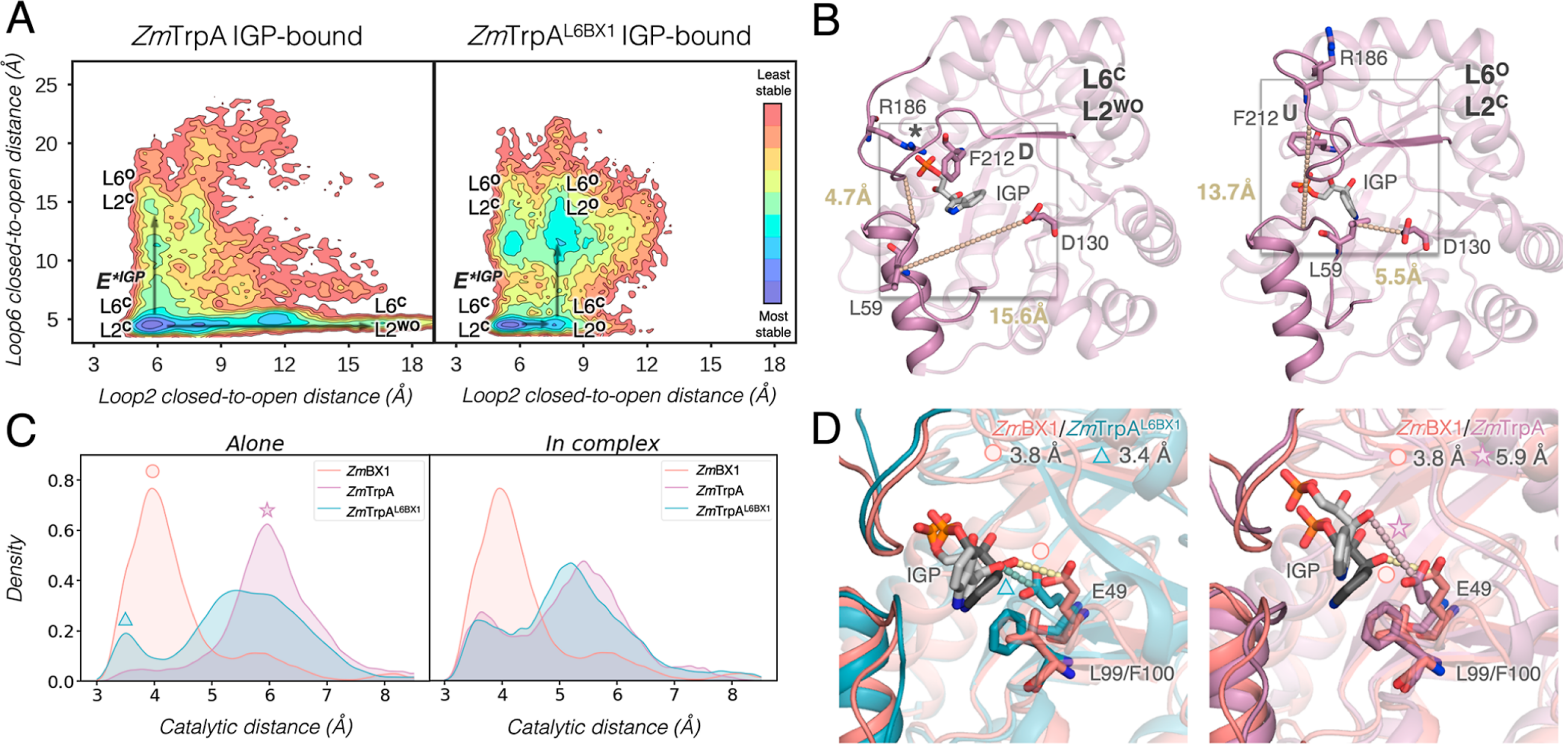
(A) Reconstructed FEL of *Zm*TrpA (left panel) and *Zm*TrpA^L6BX1^ (right panel) in the presence of the substrate IGP. For FEL reconstruction, the distance between Thr183 and Gly62 residues, that describes the closed-to-open transition of L6 (*y* axis), and the distance between Leu59 and Asp130 for L2 opening (*x* axis) are used. Most stable conformations are colored in blue, whereas the least stable ones are depicted in red. Each minimum in the FEL is labeled according to the open (O)/closed (C) conformation of L6 and L2. The catalytically activated E*^IGP^ presenting both L6 and L2 in a closed conformation is labeled as E*^IGP^**(L6**^**C**^**L2**^**C**^). (B) Representative structure of the *Zm*TrpA minima extracted from the FEL reconstructed via multiple replica MD simulations (10 replicas of 500 ns) is shown: **L6**^**C**^**L2**^**WO**^ presenting L6 closed and L2 in a wide-open conformation, and **L6**^**O**^**L2**^**C**^ with open and closed conformations of L6 and L2, respectively, are shown. The average distance for the two L6 and L2 closed-to-open distances (*y*, *x* axis in panel A) is included. The following residues are represented in sticks: Leu59 and Asp130 for L2, and the key residues for substrate binding/product release Arg186 and Phe212. The different conformations of Phe212 are marked with up (U)/down (D) to easily identify the differences in their side-chain conformation. Those conformations in which a salt bridge is established between the phosphate group of IGP and Arg186 are indicated with a star (*). (C) Histogram of the catalytic distance between Glu50 and IGP (in Å) for *Zm*BX1 (as reference, in pink), *Zm*TrpA (in purple), and *Zm*TrpA^L6BX1^ (in teal) as standalone (left panel) and in complex with *Zm*TrpB (right panel). In the histogram of the complexes, *Zm*BX1 has been included as a reference and 6 replicas of 400 ns MD simulations were run for the in-complex systems. (D) Representative structure of a catalytically productive conformation of *Zm*BX1 (taken from the peak of the histogram as marked with the dot in panel C) overlaid with either: a catalytically productive *Zm*TrpA^L6BX1^ conformation (teal, left panel) or a catalytically unproductive *Zm*TrpA conformation (purple, right panel). The most relevant residues are represented as sticks: Glu49/50, Leu99/Phe100, and IGP. The distance between Glu50 and IGP (in Å) for the displayed conformation is also included.

The rate-determining step of the TrpA reaction in the presence of TrpB is the conformational change leading to the catalytically activated E*^IGP^ state.^10,31^ We therefore hypothesized that the reduced standalone activity of TrpA might be attributed to its inability to access the activated E*^IGP^ state in the absence of TrpB. To further elucidate the importance of L6 and L2 for enhanced standalone TrpA activity, we decided to reconstruct the closed-to-open conformational landscape of *Zm*TrpA and the previously reported variant *Zm*TrpA^L6BX1^.^32^ The transfer of L6 from *Zm*BX1 to *Zm*TrpA enhances the turnover number *k*_cat_ toward IGP cleavage at the expense of worsening the Michaelis constant *K*_M_ (Table 1).^32^ Our MD simulations started from the AlphaFold2-generated models of *Zm*TrpA and *Zm*TrpA^L6BX1^ presenting L6 and L2 in a closed conformation (E* state; see the Computational Methods section in the Supporting Information). The multiple replica nanosecond time scale MD simulations confirm that in both cases, L6 can adopt the closed conformation even in the absence of IGP (minimum named E*^IGP^**(L6**^**C**^**L2**^**C**^**)** in Figure 3A and E*(L6^**C**^**L2**^**C**^**)** in Figure S4). It should be noted that this rather overestimated stability of the closed conformation might be an artifact of the MD simulations that start from the closed state (as found for *Zm*BX1 described above). However, in contrast to what is observed for *Zm*BX1, *Zm*TrpA has a rather limited conformational heterogeneity as open states of L6 are hardly sampled (only L2 adopts open states, **L6**^**C**^**L2**^**O**^, Figure S4). The transferred L6 in *Zm*TrpA^L6BX1^ favors the opening of L6, especially when L2 is also open as observed for *Zm*BX1. These simulations in the absence of IGP suggest that *Zm*TrpA^L6BX1^ can sample additional open L6 states compared to *Zm*TrpA that are of importance for IGP binding and G3P/indole release.

**Table 1:** Steady-State Kinetic Constants for *Zm*BX1, *Zm*TrpA, and Its Variants, in the Absence and Presence of *Zm*TrpB

protein	*k*_cat_ [s^–1^]	*K*_M_ [μM]	*k*_cat_/*K*_M_ [M^–1^ s^–1^]	fold activity increase^a^	fold activation^b^
*Zm*BX1	5.2 ± 0.13	11 ± 1.3	474,044 ± 56,093		
*Zm*TrpA	0.005 ± 0.001	1530 ± 327	3.3 ± 0.8		
*Zm*TrpA + *Zm*TrpB	2.9 ± 0.10	195 ± 17.9	15,006 ± 1430		4515
*Zm*TrpA^SPM4^	0.01 ± 0.0004	2286 ± 151	5.1 ± 0.4	1.5	
*Zm*TrpA^SPM4^ + *Zm*TrpB	1.0 ± 0.021	134 ± 9.0	7541 ± 522		1478
*Zm*TrpA^SPM6^	0.04 ± 0.003	2530 ± 297	14 ± 3.0	4.4	
*Zm*TrpA^SPM6^ + *Zm*TrpB	0.70 ± 0.02	103 ± 13.1	6736 ± 868		462
*Zm*TrpA^L6BX1^	1.2 ± 0.090	3351 ± 340	355 ± 45.4	108	
*Zm*TrpA^L6BX1^ + *Zm*TrpB	0.35 ± 0.013	111 ± 14.1	3106 ± 410		8.9
*Zm*TrpA^SPM4-L6BX1^	0.59 ± 0.027	1110 ± 105	533 ± 56	163	
*Zm*TrpA^SPM4-L6BX1^ + *Zm*TrpB	0.29 ± 0.013	70 ± 12	4080 ± 698		6.5

a Fold TrpA activity increase in terms of *k*_cat_/*K*_M_ of each variant alone compared to *Zm*TrpA.

b Fold activation in terms of *k*_cat_/*K*_M_ of each variant by *Zm*TrpB. Experimental conditions: 100 mM EPPS/KOH (pH 7.5), 180 mM KCl, 40 μM PLP, 6 mM NAD^+^, 20 mM Na_3_AsO_4_, 100 mM l-serine (if *Zm*TrpB was present), 5 μM GAP dehydrogenase from *Thermotoga maritima*, and varying concentrations of IGP. The reactions were performed at 30 °C. For measurements in the absence of *Zm*TrpB, 25 nM *Zm*BX1, 15 μM *Zm*TrpA, 10 μM *Zm*TrpA^SPM4^, 4.5 μM *Zm*TrpA^SPM6^, 0.5 μM *Zm*TrpA^L6BX1^, and 0.5 μM *Zm*TrpA^SPM4-L6BX1^ were used. For measurements in the complex, 0.5 μM *Zm*TrpA and 10 μM *Zm*TrpB or 0.2 μM *Zm*TrpA^SPM4^, 0.1 μM *Zm*TrpA^SPM6^, 0.1 μM *Zm*TrpA^L6BX1^, or 0.2 μM *Zm*TrpA^SPM4-L6BX1^ were used in combination with 5 μM *Zm*TrpB. The corresponding Michaelis–Menten curves are shown in Figure S6.

IGP-bound simulations for *Zm*TrpA and *Zm*TrpA^L6BX1^ using the same L6 and L2 distances (Figure 3) indicate that *Zm*TrpA presents a highly disordered L2, which adopts closed conformations but also widely open states (**L6**^**C**^**L2**^**WO**^, Figure 3A,B). L2 contains the catalytic residue Asp61 (Asp60 in *Zm*BX1 numbering), and therefore, open states of L2 are deemed detrimental for catalysis. Indeed, the analysis of the distance between Glu50 and IGP shows that catalytically productive, short distances (less than 5 Å between the carbon atom of the carboxyl group of Glu50 and the oxygen of IGP) are hardly sampled (Figure 3C, left panel). In fact, the most sampled distance is 6.0 ± 0.6 Å. This contrasts with what is observed in *Zm*BX1 that presents a shorter distance of 4.0 ± 0.4 Å (see the histogram peak in Figure 3C, left panel). In *Zm*TrpA^L6BX1^, the percentage of frames below a 5 Å threshold is increased as compared to *Zm*TrpA, but still the most visited distance is substantially longer than that for *Zm*BX1 (5.4 ± 0.7 Å, Figure 3C). This is in line with the poor Michaelis constant (*K*_M_) found experimentally for *Zm*TrpA^L6BX1^ (Table 1).^32^ The longer distances observed for both *Zm*TrpA and *Zm*TrpA^L6BX1^ can be in part attributed to the bulkier Phe100, which displaces IGP and pushes it far away from Glu49 (Figure 3D, right panel). In *Zm*BX1, Phe100 corresponds to Leu99, which is identified as a potential hotspot in the next sections (Figure 3D). The transfer of L6 in *Zm*TrpA^L6BX1^ disfavors open L2 states when IGP is bound in the active site and thus enhances the number of conformations displaying catalytically productive distances between IGP and Asp61 (Figure S3). In *Zm*BX1, the E*^IGP^**(L6**^**C**^**L2**^**C**^**)** state is the most favorable conformation when IGP is bound, but still additional partially closed **L6**^**PC**^**L2**^**O**^ and open **L6**^**O**^**L2**^**O**^ states are sampled (Figure 2B). This is not observed for *Zm*TrpA, but it is partially recovered after L6 transfer in *Zm*TrpA^L6BX1^ in line with its superior catalytic efficiency. The analysis of the catalytic distance between Glu50 and IGP indicates that in complex with *Zm*TrpB, both *Zm*TrpA and *Zm*TrpA^L6BX1^ present a substantially higher proportion of catalytically productive distances for retro-aldol cleavage (Figure 3C, right panel).

The higher standalone activity of *Zm*TrpA after L6 transfer is mainly attributed to the stabilization of the catalytically activated state (E*^IGP^**(L6**^**C**^**L2**^**C**^**)**) that is essential for IGP cleavage. However, the transfer of L6 also significantly reduces L2 flexibility and enhances L6 conformational heterogeneity after L2 opening, which is crucial for IGP binding and G3P release. In fact, TrpA has been subjected to the evolutionary constraint of retaining indole for its transfer to TrpB, which explains its inferior ability in adopting synchronized open states of L6 and L2 for facilitating G3P and indole release.

### SPM Analysis Predicts Four Additional Mutations That Enhance Standalone Activity

As explained before, the transfer of L6 into *Zm*TrpA is not enough to reach *Zm*BX1 catalytic activities or for freeing TrpA from TrpB activation. We therefore decided to apply our SPM methodology^8,33^ to identify additional mutations to enhance the standalone catalytic efficiency of *Zm*TrpA^L6BX1^ and to decrease its dependency on the allosteric activation by *Zm*TrpB. SPM is a correlation-based tool that identifies the subset of residues (located throughout the protein) presenting a higher contribution to the enzyme conformational dynamics. It requires the construction of the correlation and distance matrix from a given set of MD simulations and provides as output a 3D graph with the most conformationally relevant positions (more details are provided in the Supporting Information).^8,33^

**Figure 4:**
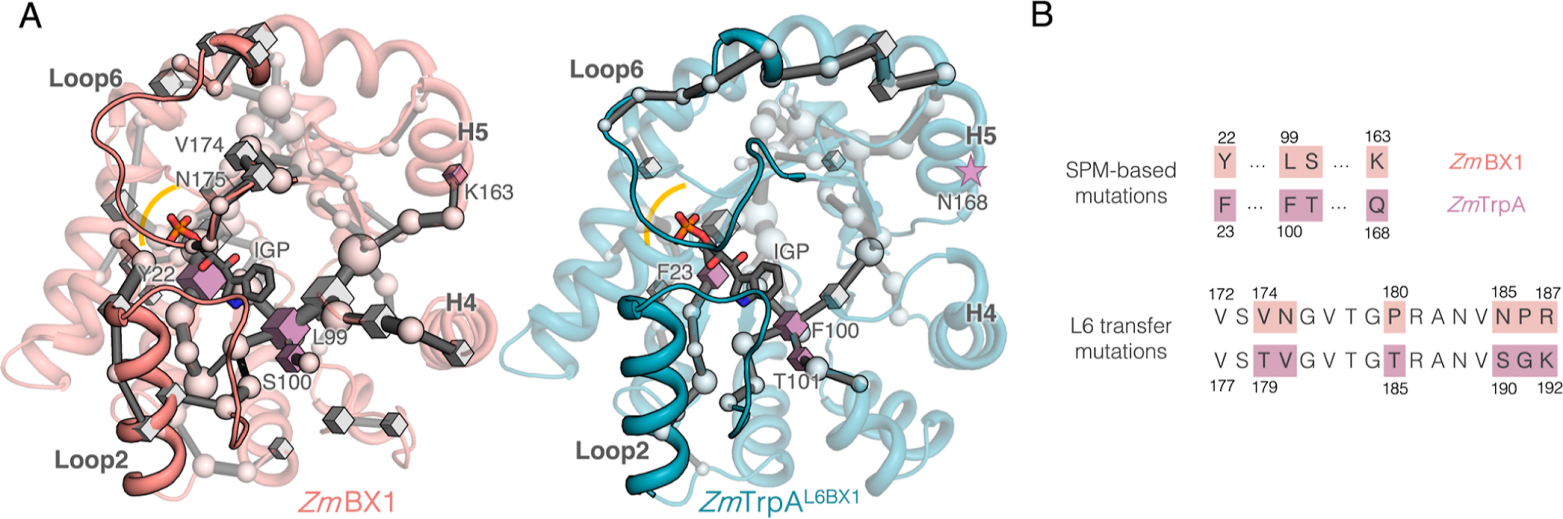
(A) Computed SPM networks for *Zm*BX1 (in pink, left) that were taken as inspiration for design, and the SPM of the starting scaffold *Zm*TrpA^L6BX1^ (in teal, right panel). Spheres are used to highlight those SPM sites that are conserved (i.e., the same amino acid is found), whereas boxes delineate nonconserved SPM positions, which correspond to potential mutational hotspots. Stars shown in the SPM of *Zm*TrpA^L6BX1^ mark the positions of the corresponding sites identified in *Zm*BX1 that are not found in *Zm*TrpA^L6BX1^. It should be noted that the nonconserved (boxes) positions that are included in L6 have also been highlighted although *Zm*TrpA^L6BX1^ contains L6 from *Zm*BX1 (i.e., the sequence comparison is made using *Zm*BX1 and *Zm*TrpA^L6BX1^). In the SPM of *Zm*BX1, the IGP phosphate-binding region (highlighted with an orange line) is connected to L2, the core of the active-site pocket where IGP is bound, and to distal areas such as α-helices H4 and H5. In the SPM of *Zm*TrpA^L6BX1^, these connections are missing, especially those with H4 and H5. Based on the SPM comparison, four mutations were predicted (highlighted with purple boxes, numbering is based on *Zm*TrpA^L6BX1^): Phe23Tyr, Phe100Leu, Thr101Ser, which are in the active site, and the distal Gln168Lys. (B) *Zm*BX1 and *Zm*TrpA sequence comparison. The nonconserved residues between *Zm*BX1 (in pink) and *Zm*TrpA (in purple) are highlighted, corresponding to SPM-based mutations (top panel) and L6 transfer mutations (bottom panel).

We generated the SPM plots for our reference *Zm*BX1 and *Zm*TrpA^L6BX1^ scaffold considering only those conformations which presented catalytically competent distances for IGP cleavage, i.e., we focused on the catalytically activated E*^IGP^**(L6**^**C**^**L2**^**C**^**)** states sampled along the IGP-bound MD simulations. The computed SPM plots for *Zm*BX1 and *Zm*TrpA^L6BX1^ including sequence conservation at the identified conformationally relevant SPM positions are shown in Figure 4A. The spheres in Figure 4A represent positions identified with SPM that are conserved between *Zm*BX1 and *Zm*TrpA^L6BX1^, whereas boxes highlight potential mutation points as both enzymes contain different amino acids at the conformationally SPM-identified sites. The SPM positions identified at L6 are also highlighted with boxes if the positions are nonconserved between *Zm*BX1 and *Zm*TrpA (i.e., positions Val174 and Asn175, see Figure 4A, left panel). Interestingly, we observed some common patterns between the computed SPMs for the two systems, such as the connection of L6 with adjacent α-helices but also some key differences. In *Zm*BX1, many residues in the phosphate binding region are identified in the SPM, and they are directly connected to L2. At the same time, L2 is connected to the core of the IGP-binding pocket and identifies the previously mentioned Leu99 as a key residue that helps in the productive binding of IGP. The network of residues in the core of the protein is also connected to more remote areas, in particular α-helices H4 and H5. Interestingly, this highly interconnected network observed for *Zm*BX1 in these three regions in *Zm*TrpA^L6BX1^ presents either a very low contribution to the SPM (core of the protein) or is not observed (the connection with the distal α-helices, and the intertwined communication between L2 and the active site, Figure 4A, right). This analysis prompted us to propose four additional mutations in *Zm*TrpA^L6BX1^ for enhancing the productive binding of IGP: Phe23Tyr, Phe100Leu, Thr101Ser, which are in the active site, and Gln168Lys, which is distant from the active site for recovering the communication of the core of the enzyme and α-helix H5. This yielded *Zm*TrpA^SPM4-L6BX1^, which we computationally and experimentally characterized. The SPM path of *Zm*TrpA^SPM4-L6BX1^ indeed confirmed that the introduced mutations successfully recovered the communication of the core region of the enzyme with α-helix H5, as well as the connection between the phosphate region and L2 (Figure S5). As shown in Table 1, the introduced mutations slightly reduce *k*_cat_ but have a positive effect on the *K*_M_, thus yielding a better *k*_cat_/*K*_M_ as compared to *Zm*TrpA^L6BX1^. In fact, in relation to *Zm*TrpA, the *Zm*TrpA^SPM4-L6BX1^ shows a 163-fold improvement in terms of *k*_cat_/*K*_M_, which is ca. 1.5-fold higher than the one obtained only after L6 transfer in *Zm*TrpA^L6BX1^. Another interesting observation is that the introduced mutations decrease the dependency on *Zm*TrpB, which enhances the activity of *Zm*TrpA^SPM4-L6BX1^ ca. 6.5-fold and ca. 8.9-fold in case of *Zm*TrpA^L6BX1^.

To further elucidate the effect of SPM mutations and role of L6 transfer, we decided to evaluate two additional variants: *Zm*TrpA^SPM4^ containing only the 4 SPM mutations identified, and *Zm*TrpA^SPM6^ including the 4 SPM mutations in the core of the protein and the two additional SPM sites contained in L6 (Thr179Val and Val180Asn). As shown in Table 1, *Zm*TrpA^SPM4^ and *Zm*TrpA^SPM6^ present a modest 4- and 7-fold improvement in *k*_cat_ and ca. 1.5- and 4.4-fold increase in *k*_cat_/*K*_M_, respectively, thus highlighting the key role of transferring L6 for higher levels of activity.

### SPM Mutations Enhance IGP Productive Binding at the Catalytically Activated E*^IGP^**(L6**^**C**^**L2**^**C**^**)** Closed State

**Figure 5:**
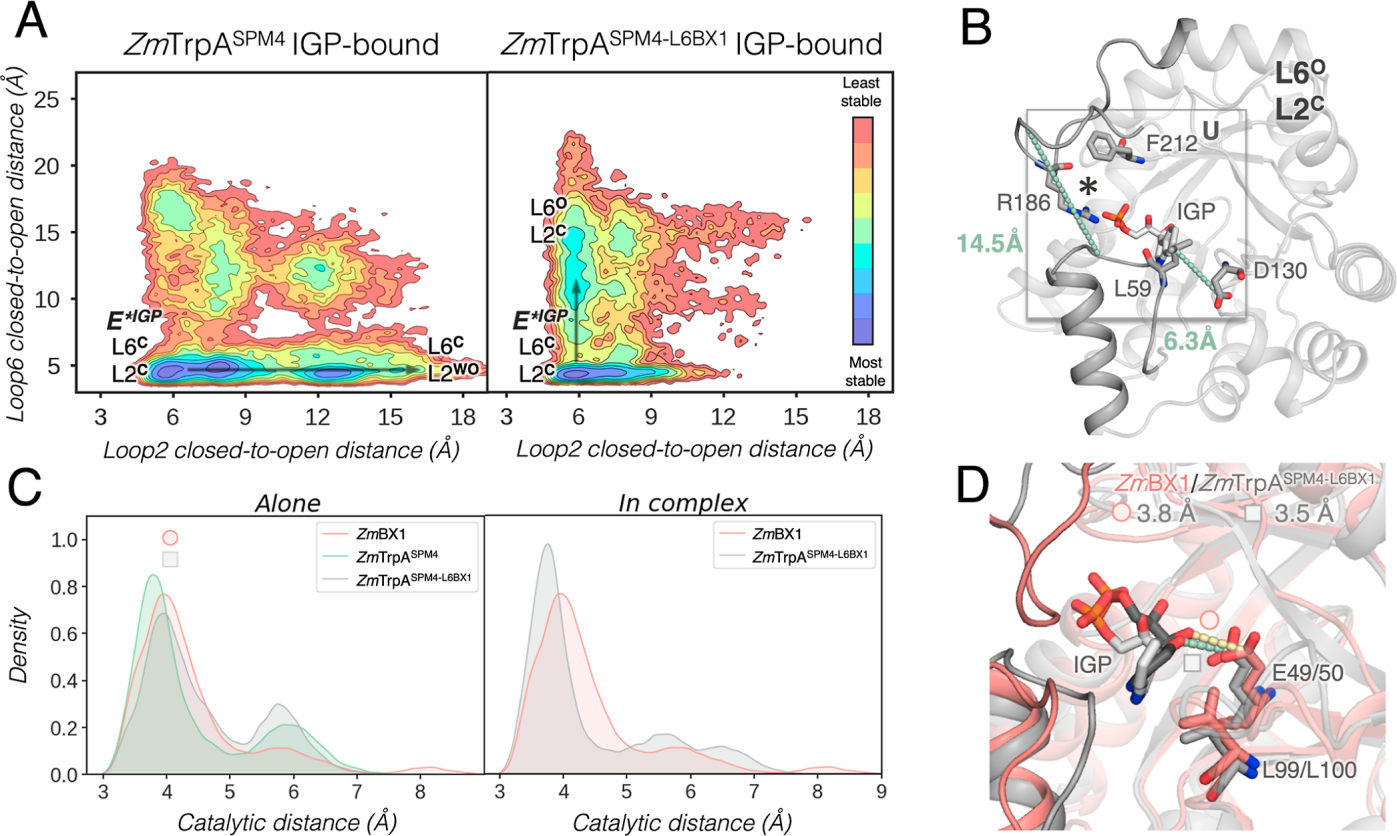
(A) Reconstructed FEL of *Zm*TrpA^SPM4^ (left panel) and *Zm*TrpA^SPM4-L6BX1^ (right panel) in the presence of the substrate IGP. For the FEL reconstruction, the distance between Thr183 and Gly62 residues, that describes the closed-to-open transition of L6 (*y* axis), and the distance between Leu59 and Asp130 for L2 opening (*x* axis) are used. Most stable conformations are colored in blue, whereas the least stable ones are depicted in red. Each minimum in the FEL is labeled according to the open (O)/closed (C) conformation of L6 and L2. The catalytically activated E*^IGP^ presenting both L6 and L2 in a closed conformation is labeled as E*^IGP^**(L6**^**C**^**L2**^**C**^). (B) Representative structure of the *Zm*TrpA^SPM4-L6BX1^ minimum extracted from the FEL reconstructed via multiple replica MD simulations (10 replicas of 500 ns): **L6**^**O**^**L2**^**C**^ presenting L6 open and L2 closed is shown. The average distance for the two L6 and L2 closed-to-open distances (*y*, *x* axis in panel A) is included. The following residues are represented in sticks: Leu59 and Asp130 for L2, and the key residues for substrate binding/product release Arg186 and Phe212. The different conformations of Phe212 are marked with up (U)/down (D) to easily identify the differences in their side-chain conformation. The established salt bridge between the phosphate group of IGP and Arg186 is indicated with a star (*). (C) Histogram of the catalytic distance between Glu50 and IGP (in Å) for *Zm*BX1 (as reference, in pink), *Zm*TrpA^SPM4^ (green), and *Zm*TrpA^SPM4-L6BX1^ (in gray) as standalone (left panel) and in complex with TrpB (right panel). In the histogram of the complexes, *Zm*BX1 has been included as a reference and 6 replicas of 400 ns MD simulations were run for the in-complex systems. (D) Representative structure of a catalytically productive conformation of *Zm*BX1 (taken from the peak of the histogram as marked with the dot in panel C) overlaid with a catalytically productive *Zm*TrpA^SPM4-L6BX1^ conformation (gray, left panel). The most relevant residues are represented as sticks: Glu49/50, Leu99/Leu100, and IGP. The distance between Glu50 and IGP (in Å) for the displayed conformation is also included.

The evaluation of the conformational landscape of the new *Zm*TrpA^SPM4^ and *Zm*TrpA^SPM4-L6BX1^ variants in the presence of IGP (Figure 5A,B) shows that the four SPM additional mutations are not able to restrict the L2 flexibility. *Zm*TrpA^SPM4^ containing only the SPM mutations in the core of the enzyme and at distal sites (none at L6) displays a reduced stability of E*^IGP^**(L6**^**C**^**L2**^**C**^**)**, as additional conformations with L2 in open conformations (i.e., **L6**^**C**^**L2**^**O**^, Figure 5A) are also visited. These results suggest that L6 or additional mutations at L6 are needed to decrease the flexibility of L2 and stabilize the catalytically activated E*^IGP^**(L6**^**C**^**L2**^**C**^**)** state for enhancing the standalone activity. Interestingly, the open states of L2 are substantially destabilized in *Zm*TrpA^SPM6^ (Figure S7), which has the two additional SPM mutations identified in L6 (Thr179Val and Val180Asn). The transfer of L6 in *Zm*TrpA^SPM4-L6BX1^ stabilizes the catalytically activated E*^IGP^**(L6**^**C**^**L2**^**C**^**)** state as shown in Figure 5B, in line with its superior standalone catalytic activity. However, one of the limitations of *Zm*TrpA^SPM4-L6BX1^ as compared to *Zm*BX1 is that the catalytically activated E*^IGP^**(L6**^**C**^**L2**^**C**^**)** minimum is much broader as it presents closed-to-open L2 distances ranging between 5 and 8 Å (for *Zm*BX1, it ranges between 4 and 6 Å, see Figures 2B and 5B). Another difference is the inability of *Zm*TrpA^SPM4-L6BX1^ to visit the open states of both L6 and L2 (i.e., **L6**^**O**^**L2**^**O**^, Figure 5B). Instead, this variant can explore a new conformation not observed for *Zm*BX1 in which L6 is in an open state, while L2 remains closed (i.e., **L6**^**O**^**L2**^**C**^, Figure 5B). In **L6**^**O**^**L2**^**C**^, Arg186 establishes the previously mentioned salt bridge with the phosphate group of IGP for promoting G3P release after the retro-aldol reaction. However, the closed conformation of L2 most likely hampers the final indole release after IGP cleavage. The number of frames presenting proper catalytic distances between Glu50 and IGP at the **L6**^**C**^**L2**^**C**^ state is drastically enhanced as compared to those of *Zm*TrpA and *Zm*TrpA^L6BX1^ (Figure 5C, left panel), which altogether is in line with the higher catalytic efficiency of the new *Zm*TrpA^SPM4-L6BX1^ variant. As shown in Figure 5D, the representative IGP-bound conformation obtained for *Zm*TrpA^SPM4-L6BX1^ perfectly matches the one observed for *Zm*BX1. One of the key mutations for the enhancement of the catalytic IGP–Glu50 distance is Phe100Leu. This mutation provides additional space for the indole ring of IGP and allows it to stay closer to catalytic Glu50, which is also observed in *Zm*BX1. This translates to a higher proportion of frames with productively bound IGP, thus, yielding a substantially better *K*_M_ value. Despite presenting proper catalytic distances for IGP cleavage, the *k*_cat_ value for *Zm*TrpA^SPM4-L6BX1^ is lower than that for *Zm*TrpA^L6BX1^ (Table 1). Our calculations therefore suggest that the smaller *k*_cat_ observed in *Zm*TrpA^SPM4-L6BX1^ as compared to *Zm*TrpA^L6BX1^ is mostly attributed to the much wider range of closed-to-open L2 distances sampled at the E*^IGP^**(L6**^**C**^**L2**^**C**^**)** state: 5–8 Å in *Zm*TrpA^SPM4-L6BX1^, compared to 5–6.2 Å in *Zm*TrpA^L6BX1^ (in *Zm*BX1, it is 4–6 Å, Figure 2B). This difference in terms of the L2 distance adopted at the catalytically activated E*^IGP^ state is crucial for catalysis, as it directly affects the catalytically relevant Asp60–IGP distance (Figure S3). We performed density functional theory calculations to evaluate the impact of L2 conformation (i.e., the effect of the Asp60–IGP distance) for the stabilization of the reactant complex and found that the L2 closed state is favored by ca. 3.3 kcal/mol (Figure S8). Altogether these calculations suggest that high levels of standalone activity require the stabilization of the E*^IGP^**(L6**^**C**^**L2**^**C**^**)** state, presenting L2 closed distances ranging between 4 and 6 Å, with larger values being highly detrimental for catalysis. The comparison with *Zm*BX1 also indicates that simultaneously adopting open states of both L6 and L2 at the IGP-bound state is also important for favoring IGP binding and G3P/indole release after retro-aldol cleavage.

The number of catalytically productive frames of *Zm*TrpA^SPM4-L6BX1^ in complex with TrpB is only slightly increased, as compared to the values obtained in isolation (Figure 5C, right panel). This is in line with the substantially lower activation observed experimentally (in complex, the catalytic efficiency is enhanced <7-fold, see Figure 4B and Table 1). This contrasts with the substantial increase in catalytically productive distances observed in the case of *Zm*TrpA, which is activated 4515-fold by *Zm*TrpB and *Zm*TrpA^SPM4^, which is activated 1478-fold (Table 1).

## Conclusions

TrpA and its standalone homologue *Zm*BX1 share a high structural similarity. However, they display dramatically different conformational dynamics, especially of catalytically relevant active-site loops L6 and L2. *Zm*BX1 can adopt closed and open states of both L6 and L2 in the absence of any ligand: closed states are important for catalysis, as they properly position the catalytic residues Glu49/Glu50 and Asp60/Asp61, whereas open states play a key role in substrate binding and product release. In the presence of IGP, the catalytically activated E*^IGP^ state presenting both L6 and L2 in a closed conformation is stabilized, but still *Zm*BX1 can open L2, which in turn initiates/enables L6 opening, thus enhancing product release after retro-aldol cleavage. In fact, in these open states, we find a key contribution of Arg181, previously hypothesized to be essential for faster kinetics of TrpA,^10^ which establishes a salt bridge with the phosphate group of IGP and might promote IGP binding and G3P release after completion of the reaction. This interplay between L6 and L2 dynamics is completely missing in *Zm*TrpA in the absence of TrpB but is partially recovered after incorporating L6 of *Zm*BX1 into *Zm*TrpA. The previously reported *Zm*TrpA^L6BX1^ variant^32^ displays a higher catalytic efficiency due to the restriction of L2 dynamics and enhancement of L6 flexibility when L2 adopts open states.

Considering that the rate-determining step of the TrpA reaction in the presence of serine and TrpB is the conformational transition to reach the catalytically activated E*^IGP^ state,^10,31^ we evaluated how the network of intramolecular pathways differs between the *Zm*TrpA^L6BX1^ starting scaffold and the reference *Zm*BX1 at the catalytically activated E*^IGP^ state using our correlation-based method SPM. Our analysis suggested that four additional mutations were required to stabilize the catalytically activated E*^IGP^ state with both L6 and L2 closed to enhance the productive binding of IGP and to recover the intramolecular pathway observed in *Zm*BX1. Although the newly generated variant *Zm*TrpA^SPM4-L6BX1^ does not fully recover *Zm*BX1’s ability to visit open states of L6 and L2, it can perfectly bind IGP in the same conformation as *Zm*BX1 and can substantially stabilize the catalytically activated E*^IGP^ state, thus decisively enhancing the overall catalytic efficiency by 163-fold. This improvement is interestingly similar to the previously reported 150-fold enhancement of the rate for IGP cleavage at TrpA induced by the formation of the E(A–A) intermediate at TrpB in the presence of serine.^31^ When E(A–A) is formed at TrpB, the conformational transition of TrpA to the catalytically activated state E*^IGP^ is favored.^31^ Although in previous studies the conformational change leading to the catalytically activated E*^IGP^ was hypothesized to be the open-to-closed transition of L6,^10^ our study indicates that it also involves L2, being the catalytically activated E*^IGP^ state, the one presenting both L6 and L2 in a closed conformation. This closed catalytically activated E*^IGP^ state is crucial for catalysis but also for retaining and channeling indole to the TrpB subunit in the physiological process of l-Trp synthesis. Our study demonstrates that the stabilization of the closed catalytically activated E*^IGP^ state is required for standalone TrpA activity; however, a synchronized L6/L2 dynamics is also needed for accessing open states of importance for IGP binding and G3P/indole release. MD simulations indicate that in *Zm*BX1, the aperture of L2 favors the opening of L6; however, in *Zm*TrpA and the variants displaying low standalone activity, open states of L6 mostly present L2 closed. While our work focuses on TrpA engineering, we expect that the developed SPM-based methodology can be broadly applied, especially in enzymes where conformational change is the rate-determining factor.

## Supporting Information Available

Computational methods and experimental protocols (PDF)
