# Supplementary material for: Altering Active-Site Loop Dynamics Enhances Standalone Activity of the Tryptophan Synthase Alpha Subunit
Source: ACS Catal. 2024 Nov 2;14(22):16986–95. doi: 10.1021/acscatal.4c04587 (PMC11574760; doi:10.1021/acscatal.4c04587)
Supplement: Supplementary file 1 — cs4c04587_si_001.pdf [file cs4c04587_si_001.pdf]

Altering Active Site Loop Dynamics Enhances Stand-Alone Activity of Tryptophan Synthase alpha-Subunit

Cristina Duran,[a] Thomas Kinateder,[b] Caroline Hiefinger,[b] Reinhard Sterner,*[b] and Sílvia Osuna*[a,c]

[a] Cristina Duran and Prof. Sílvia Osuna
Institut de Química Computacional i Catàlisi and Departament de Química
c/ Maria Aurèlia Capmany 69, 17003 Girona, Spain
E-mail: silvia.osuna@udg.edu

[b] Dr. Thomas Kinateder, Caroline Hiefinger, Prof. Reinhard Sterner
Institute of Biophysics and Physical Biochemistry, Regensburg Center for Biochemistry
University of Regensburg
D-93040 Regensburg, Germany
E-Mail: reinhard.sterner@ur.de

[c] Prof. Sílvia Osuna
ICREA
Pg. Lluís Companys 23, 08010 Barcelona, Spain

SUPPORTING INFORMATION

Computational Methods:

Molecular modeling system preparation. The starting structures for the seven systems (*Zm*BX1, *Zm*TrpA, *Zm*TrpS, *Zm*TrpA^{L6BX1}, *Zm*TrpS^{L6BX1}, *Zm*TrpA$^{SPM4-L6BX1}$, *Zm*TrpS$^{SPM4-L6BX1}$) were generated with the multimer version of the AlphaFold2 (AF2)[1] neural network. The AF2 models simulated had a predicted LDDT-Cα score (pLDDT) higher than 92. To generate the version of the isolated TrpA systems, the alpha subunit from the TrpS model was extracted. For the homodimeric *Zm*BX1 enzyme, two X-ray structure are available (PDB codes: 1TJR and 1RD5) corresponding to two different dimerization modes. All *Zm*BX1 models generated were predicted to have the 1RD5 dimerization mode, thus it was used for running Molecular Dynamics (MD) simulations. The IGP substrate was placed in the TrpA subunits both in the isolated TrpA and the TrpA construct in complex through superposition to the G3P product in the Last Bacterial Common Ancestor TrpS X-ray structure (PDB code: 5EY5). For the heterocomplex MD simulations, the amino acrylate intermediate (A-A) was superimposed onto the same intermediate present in the *Salmonella typhimurium* TrpS X-ray structure (PDB code: 2J9X).

The water molecules added to each subunit were selected from the DBSCAN clusterization[2, 3] algorithm implemented in the scikit-learn Python library.[4] For TrpA, the water molecules of different TrpA subunits were used, ordered by PDB accession number and chain: 1TJR_A, 1TJR_B, 1RD5_A, 1RD5_B, 5EY5_A, 5EY5_C, 2DZS_A, 2DZS_B, 2DZU_A, 2DZU_B, 2E09_A, 2E09_B, 2DZW_A, 2DZW_B, 1GEQ_A, 1GEQ_B, 5E0K_A, 5E0K_C, 5E0K_E, 5E0K_G, 5E0K_I, 5E0K_K, 1WDW_A, 1WDW_C, 1WDW_E, 1WDW_G, 1WDW_I, 1WDW_K, 2DZT_A, 2DZT_B, 2DZV_A, 2DZV_B, 2DZP_A, 2DZP_B, 2DZX_A, 2DZX_B. In the MD simulations run on the IGP-bound state, the water molecules clashing with the substrate were removed manually. For the heterodimeric MD simulations, the water molecules of the following TrpB subunits were used, ordered by PDB accession number and chain: 5IXJ_A, 5IXJ_B, 5IXJ_C, 5IXJ_D, 5DW3_A, 5DW3_B, 5DW3_C, 5DW3_D, 5E0K_B, 5E0K_D, 5E0K_F, 5E0K_H, 5E0K_J, 5E0K_L, 5DW0_A, 5DW0_B, 5DW0_C, 5DW0_D, 1V8Z_A, 1V8Z_B, 1V8Z_C, 1V8Z_D, 5T6M_A, 5T6M_B, 5T6M_C, 5T6M_D, 1WDW_B, 1WDW_D, 1WDW_F, 1WDW_H, 1WDW_J, 1WDW_L, 5DVZ_A, 5DVZ_B, 5DVZ_C, 5DVZ_D, 6AMH_A, 6AMH_B, 6AMH_C, 6AMH_D, 6AMI_A, 6AMI_B, 6AMI_C, 6AMI_D, 6AMC_A, 6AMC_B, 6AMC_C, 6AMC_D, 5VM5_A, 5VM5_B, 5VM5_C, 5VM5_D, 6AM8_A, 6AM8_B, 6AM8_C, 6AM8_D, 6AM9_A, 6AM9_B, 6AM9_C, 6AM9_D, 6AM7_A, 6AM7_B, 6AM7_C, 6AM7_D, 6CUV_A, 6CUV_B, 6CUV_C, 6CUV_D, 6CUT_A, 6CUT_B, 6CUT_C, 6CUT_D, 6CUZ_A, 6CUZ_B, 6CUZ_C, 6CUZ_D, 5EY5_B, 5EY5_D. Additionally, one conserved sodium ion in the X-ray structure was added to the TrpB subunit located close to the active site.

The MD parameters for the substrate IGP and the A-A intermediate were generated with the antechamber and parmchk2 modules of AMBER20[5] using the 2nd generation of the general amber force-field (GAFF2).[5, 6] The IGP substrate and A-A intermediate were optimized at the B3LYP/6-31G(d) level of theory including Grimme's dispersion correction with Becke-Johnson Damping (D3-BJ) and the polarizable conductor model (PCM) (diethyl ether, $\varepsilon = 4.2$) as an estimation of the dielectric permittivity in the enzyme active site.[7] The partial charges (RESP model)[8] were set to fit the electrostatic potential generated at the HF/6-31G(d) level of theory. The charges were calculated according to the Merz-Singh-Kollman[9] scheme using the Gaussian16 software package.[10] The protonation states were predicted using PROPKA.[11, 12] For *Zm*BX1, *Zm*TrpA, *Zm*TrpA^{L6BX1} and *Zm*TrpA$^{SPM4-L6BX1}$ the protonation state of the catalytic

residue Glu49/50 was neutral (i.e., GLH49 and GLH50), as is described in the TrpA mechanism. For the heterocomplex simulations, the protonation state of the TrpB catalytic residue Lys84 was neutral (i.e., LYN84), as is described in the mechanism.[13] The enzyme structures were solvated in a pre-equilibrated truncated octahedral box of 10 Å edge distance using the OPC water model and neutralized by the addition of explicit counterions (i.e., Na^+) using the AMBER20 leap module. All MD simulations were performed using a modification of the amber99 force field (ff19SB).[14]

MD simulation details. MD equilibration phase was done following the protocol described by Roe and Brooks with small differences fine-tuned to our systems.[15] The bonds involving hydrogen are constrained by the SHAKE algorithm during the non-minimization steps. Long-range electrostatic effects were modeled using the particle mesh-Ewald method.[16] For Lennard–Jones and electrostatic interactions, a 10 Å cut-off was applied. The MD protocol starts with the minimization phase of 1500 steps of the steepest descent method followed by 3500 steps of the conjugate gradient method with a positional restrain (*i.e.*, a force constant of 5.0 $kcal{\cdot}mol^{-1}{\cdot}Å^{-2}$) to the protein heavy atoms. In the following heating phase a temperature increment from 25 K to 300K during 20 ps of MD simulation time, a Langevin thermostat with a collision frequency of 5 ps^{-1}, and a positional restrain (i.e., a force constant of 5.0 $kcal{\cdot}mol^{-1}{\cdot}Å^{-2}$) to the protein heavy atoms; are performed. A minimization and heating of all atoms in the system is the following step. This starts with two minimization stages of 1000 steps of the steepest descent method followed by 1500 steps of the conjugate gradient method each with a positional restrain (i.e., force constant of 2.0 $kcal{\cdot}mol^{-1}{\cdot}Å^{-2}$ in the first minimization and 0.1 $kcal{\cdot}mol^{-1}{\cdot}Å^{-2}$ in the second) to the protein heavy atoms. Following, a third minimization phase of 1500 steps of the steepest descent method followed by 3500 steps of the conjugate gradient method without any positional restraint is performed. The system is then heated in accordance with the previously established procedure. Finally, a five-round equilibration phase at the NPT ensemble with a constant pressure of 1 atm is performed. The first four rounds were done with the Berendsen barostat, whereas the fifth one was done with a Monte-Carlo barostat. For all equilibration rounds, Langevin thermostat with a collision frequency of 1 ps^{-1} was used. A positional restraint to the protein-heavy atoms with a force constant of 1.0 and 0.5 $kcal{\cdot}mol^{-1}{\cdot}Å^{-2}$ was applied to the first and second equilibration rounds, respectively. In the third round of 10 ps equilibration, a positional restraint to the backbone-heavy atoms with a force constant of 0.5 $kcal{\cdot}mol^{-1}{\cdot}Å^{-2}$ was used. The fourth and fifth equilibration of 10 ps and 1 ns, respectively, were performed without any restraint. The production runs were performed at the NVT ensemble with the Langevin thermostat with a collision frequency of 1 ps^{-1} during 500 ns for all TrpA and *Zm*BX1 systems, and 400 ns for TrpS complexes. A total of 10 replicas of equilibration and production runs were performed reaching a total simulation time of 5 µs/system (10 replicas x 500 ns) for *Zm*BX1, *Zm*TrpA, *Zm*$TrpA^{L6BX1}$, *Zm*$TrpA^{SPM4}$, *Zm*$TrpA^{SPM6}$ and *Zm*$TrpA^{SPM4\text{-}L6BX1}$ systems. For the heterocomplexes (*i.e.*, *Zm*TrpS, *Zm*$TrpS^{L6BX1}$ and *Zm*$TrpS^{SPM4\text{-}L6BX1)}$) 6 replicas of equilibration and production runs were performed reaching a total simulation time of 2.4 µs for each system (6 replicas x 400 ns). The MD trajectories were analyzed using the Python packages MDTraj,[17] pytraj[18] which is part of the cpptraj package,[15] MDAnalysis,[19] and PyEMMA.[20]

Free Energy Landscape (FEL) reconstruction. Molecular dynamics (MD) simulations allow the sampling of the population distribution of biomolecules by integrating Newton's laws of motion. This process enables the recovery of thermodynamic properties such as the free energy. However, due to the vast number of atoms involved in the MD simulations, this

probability distribution of molecular states is represented in an extremely high-dimensional space. This is usually solved by focusing on a selected set of degrees of freedom (DOF) relevant to the process of interest. In our case we used the distance between Thr178 and Gly61 for the closed-to-open transition of L6 (y axis in all Figures), and the distance between Tyr58 and Asp125 for the closed-to-open transition of L2 (x axis). High dimensional data obtained from MD simulations can be projected onto these DOFs for obtaining the probability distributions and reconstructing the free energy (eq. 1).

$$G \sim -k_B T \log (P) \qquad \text{(eq. 1)}$$

where the free energy (G) is defined as the negative logarithm of the population distribution (P) in $k_B T$ units (e.g. kcal/mol·K). A maximum in the distribution corresponds to a minimum in the free energy surface.

Shortest Path Map (SPM) calculations. The Shortest Path Map (SPM) analysis was performed using the MD simulations of *Zm*BX1, *Zm*TrpA, *Zm*TrpA^{L6BX1}, *Zm*TrpA$^{SPM4\text{-}L6BX1}$ systems. For SPM calculation, the MD simulations are used to compute the inter- residue mean distance and correlation matrices. A simplified graph is created using both matrices, in which only the pairs of residues that show a mean distance of less than 6 Å along the MD simulation are connected through a line. The edge connecting both residues is weighted to the Pearson correlation value (d_{ij}=-log $|C_{ij}|$). The residues with more correlated motions, will be connected through a shorter line. The generated graph is further simplified to identify the shortest path lengths. Following this strategy, the residues whose lines in the graph are shorter (*i.e.*, with more correlated movements) and thus, play an important role in the conformational dynamics of an enzyme, are detected. Finally, the generated SPM graph is drawn onto the 3D structure of the enzyme. More details about our SPM tool can be found in reference [21] and [22].

Quantum Mechanical (QM) calculations. Geometry minimizations were performed using Gaussian16[10], using the dispersion-corrected[23] hybrid density functional theory method B3LYP-D3(BJ) functional[24, 25], and the 6-31G(d,p) basis set. All energies were calculated by performing single-point calculations on the optimized B3LYP-D3(BJ)/6-31G(d,p) geometries using the functional wB97XD[26] with the 6-311+G(2d,2p) basis set.

Experimental Methods:

Cloning. The gene for *Zm*TrpA$^{SPM4\text{-}L6BX1}$ was codon-optimized for expression in *E. coli* and synthesized by ThermoFisher Scientific (GeneArt Strings DNA Fragments). Applying GoldenGate cloning with *Bsa*I as restriction enzyme,[27] the gene was cloned into a pET21a_BsaI vector. Plasmids encoding the genes for *Zm*BX1, *Zm*TrpA, *Zm*TrpA^{L6BX1}, *Zm*TrpB were taken from previously published work.[28]

Expression and purification of recombinant proteins. *Zm*BX1, *Zm*TrpA, *Zm*TrpA^{L6BX1}, and *Zm*TrpB were produced by heterologous gene expression in *E. coli* BL21 (DE3) gold as described previously.[28] For the expression of *Zm*TrpA$^{SPM4\text{-}L6BX1}$ cells were transformed with the

respective plasmid. The cells were grown in LB medium supplemented with 150 mg/ml ampicillin at 37 °C. As soon as a cell density of OD_{600} of 0.6 was reached, protein expression was induced by addition of 0.5 mM isopropyl-β-thiogalactopyranoside (IPTG), and the cells were further incubated overnight at 20 °C. The cells were harvested by centrifugation and suspended in 50 mM Tris (pH 7.5), 300 mM NaCl, 10 mM imidazole. Cells were disrupted by sonication (Branson Sonifier W-250D; amplitude 50 %; 2 min, 30 s pulse/30 s pause) and the insoluble fraction was removed by centrifugation. The target protein was purified from the soluble fraction by immobilized metal ion affinity chromatography (IMAC, HisTrapTM FF crude or HisTrap excel, 5 mL, GE Healthcare) applying a linear imidazole gradient (10 mM to 500 mM). Fractions containing the target protein were pooled and subjected to size exclusion chromatography (SEC, GE Healthcare, HiLoad 26/600 Superdex 75 PG) using 50 mM Tris (pH 7.5), 300 mM NaCl. The purified proteins were dripped in liquid nitrogen and stored at −70 °C.

Steady-state enzyme kinetics.
Steady-state enzyme kinetics of *Zm*BX1 and *Zm*TrpA and its variants in absence and presence of *Zm*TrpB were measured at 30 °C. The cleavage of IGP to GAP and indole was monitored spectrophotometrically (JASCO V-750) at 340 nm by a coupled assay with GAP dehydrogenase [$\varepsilon_{340}(NADH\text{-}NAD^+) = 6.22\ mM^{-1}cm^{-1}$].[29] Reaction conditions included 100 mM EPPS/KOH (pH 7.5), 180 mM KCl, 40 µM PLP, 6 mM NAD^+, 20 mM Na_3AsO_4, 100 mM L-serine (if *Zm*TrpB was present), 5 µM GAP dehydrogenase from *Thermotoga maritima*, and varying concentrations of IGP. The reaction was started by the addition of either *Zm*BX1 or *Zm*TrpA variants alone or in complex with *Zm*TrpB. The amount of *Zm*TrpB that was required to reach full saturation for each *Zm*TrpA variant was determined by activity titrations beforehand. Enzyme activity was deduced from the initial slopes divided by the enzyme concentration ($[E]_0$) and plotted against the substrate concentration [S]. The catalytic parameters k_{cat} and K_M were determined by fitting the data to the Michaelis-Menten equation (1) using Origin 2021 (OriginLab).

$$\frac{v}{[E]_0} = \frac{k_{cat}\,[S]}{K_M + [S]}$$

For *Zm*TrpA^{L6BX1} and *Zm*TrpA^{SPM6} the applied substrate concentrations were not sufficient to reach saturation. Therefore, the k_{cat}/K_M value from the hyperbolic fit was confirmed employing a linear fit to the first five data points using Origin 2021 (OriginLab).

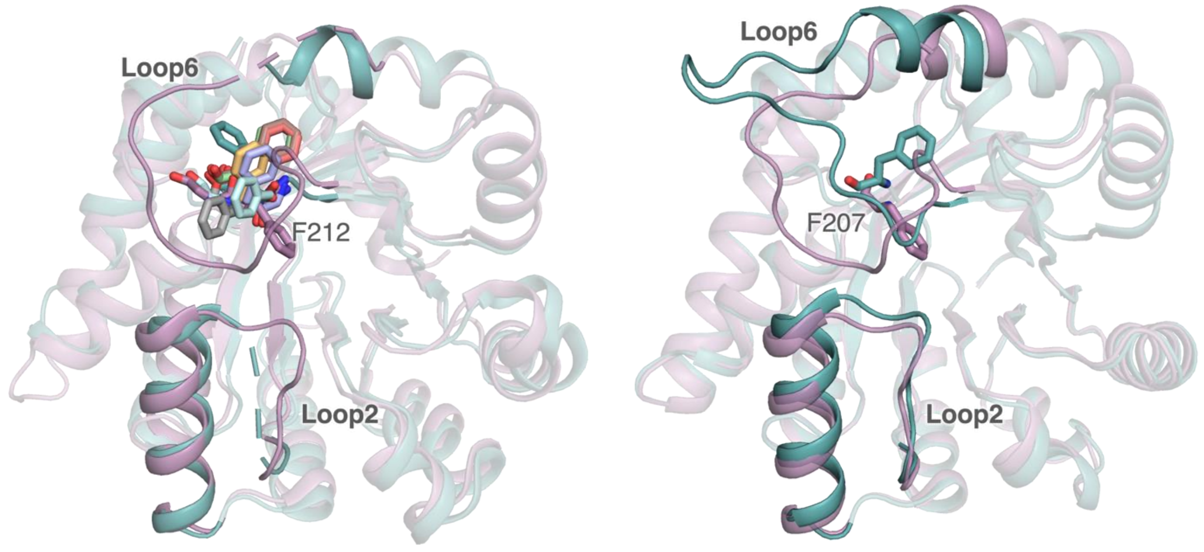

Figure S1. Overlay of different available X-ray structures of TrpA (left panel) and *Zm*BX1 (right panel). F207/212 is represented as sticks. Different colors are used to distinguish the different X-ray structures. For clarity, the cartoon is only shown for the X-rays with F207/212 in *down* conformation (violet) and in *up* conformation (teal). The PDB codes used for TrpA overlay are: 3VND, 1WXJ (*up* conformation), 6V82, 5EY5 (*down* conformation), 1GEQ, 1WDW, 5K9X, 5KMY, 2EKC, 5E0K; those correspond to all wild-type TrpAs with a sequence identity higher than 36% with *Zm*TrpA. The PDB codes for available *Zm*BX1 structures used here are: 1TJR (*down* conformation) and 1RD5 (*up* conformation).

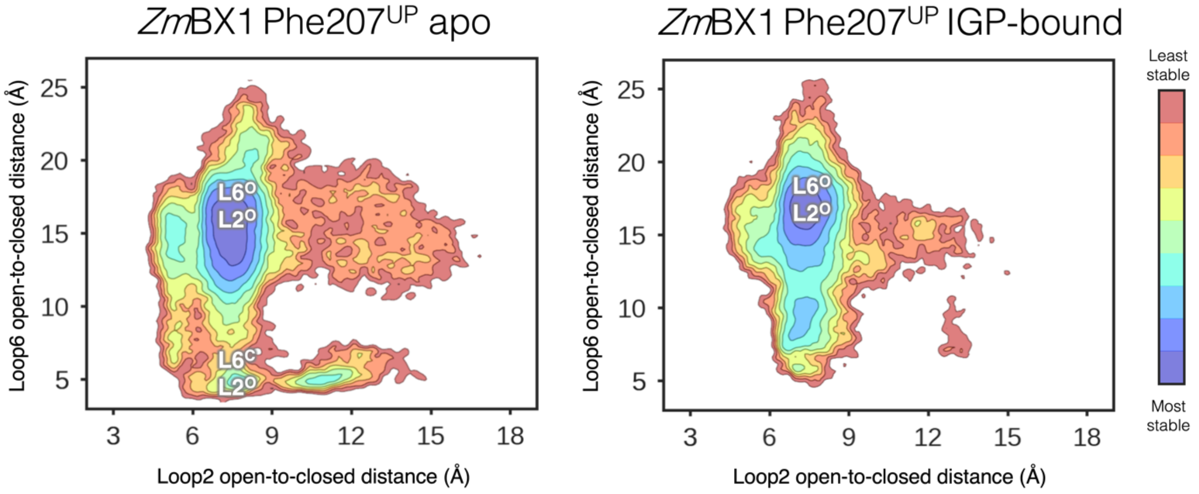

Figure S2. Reconstructed Free Energy Landscape (FEL) of *Zm*BX1 starting from Phe207 in the *up* conformation and loop 6 open in the apo state (left panel) and in the presence of the

substrate IGP (right panel). For FEL reconstruction, the distance between Thr178 and Gly61 residues that describe the open-to-closed transition of L6 (y axis), and the distance between Tyr58 and Asp125 for L2 opening (x axis) are used. Most stable conformations are colored in blue, whereas the least stable ones are depicted in red. Each minimum in the FEL is labelled according to the open (O)/closed (C) conformation of L6 and L2.

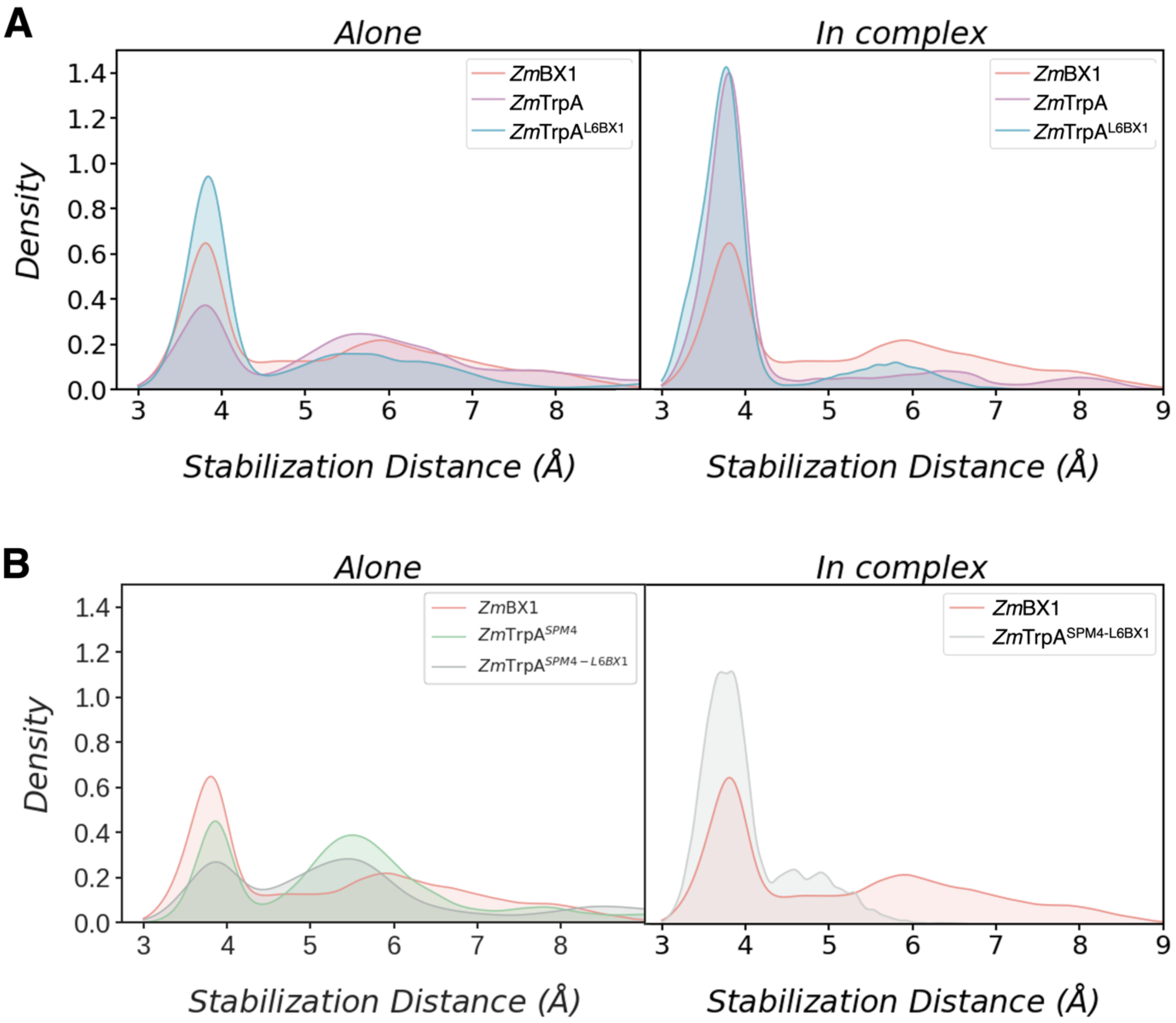

Figure S3. Histogram of the catalytic stabilization distance between Asp60/61 and IGP (in Å) for: **A** *Zm*BX1 (as reference, in pink), *Zm*TrpA (in purple), and *Zm*TrpA^{L6BX1} (in teal) and for **B** *Zm*BX1 (as reference, in pink), *Zm*TrpA^{SPM4} (in green), and *Zm*TrpA$^{SPM4\text{-}L6BX1}$ (in grey) as stand-alone (left panels) and in complex with *Zm*TrpB (right panels). In the histogram of the complexes, *Zm*BX1 has been included as a reference.

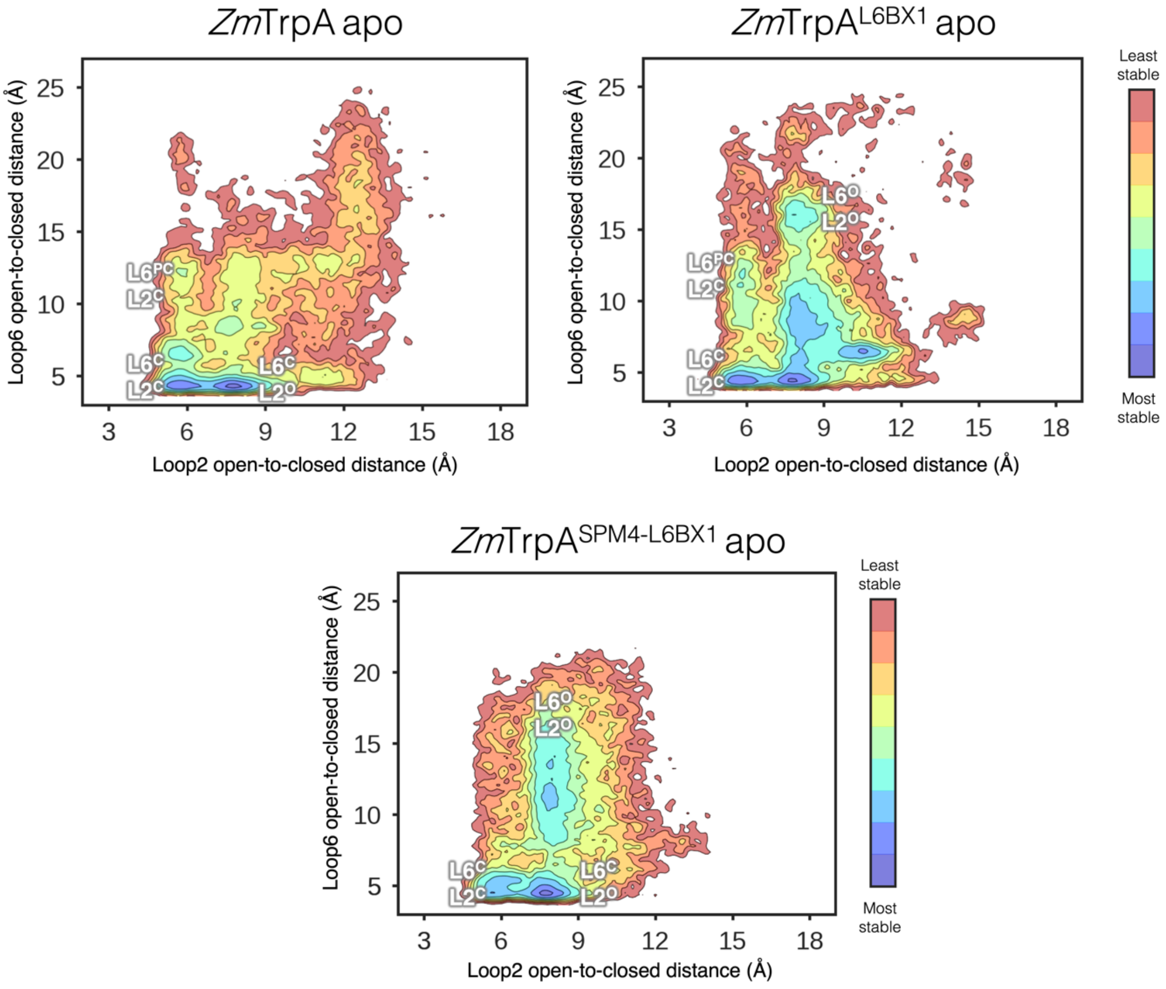

Figure S4. Reconstructed Free Energy Landscape (FEL) of *Zm*TrpA (top left panel), *Zm*TrpA^{L6BX1} (top right panel) and *Zm*TrpA$^{SPM4\text{-}L6BX1}$ (bottom panel) in the apo state. For FEL reconstruction the distance between Thr183 and Gly62 residues that describe the open-to-closed transition of L6 (y axis), and the distance between Leu59 and Asp130 for L2 opening (x axis) are used. Most stable conformations are colored in blue, whereas the least stable ones are depicted in red. Each minimum in the FEL is labelled according to the open (O)/closed (C) conformation of L6 and L2.

The evaluation of the conformational landscape of the new *Zm*TrpA$^{SPM4\text{-}L6BX1}$ variant in the absence of IGP (Figure S4, bottom panel) shows how the four additional mutations restrict L2 flexibility and allow the opening of L6, especially when L2 adopts the open state (*i.e.,* they favor the transition from **L6^{C}L2^{O}** to **L6^{O}L2^{O}**) as observed for *Zm*BX1. When IGP is bound to the active site, the catalytically productive **L6^{C}L2^{C}** state is also stabilized (Figure 5A, right).

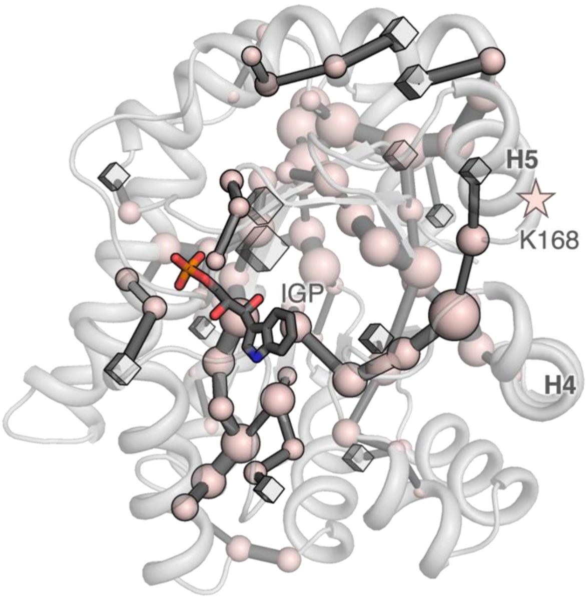

Figure S5. Shortest Path Map (SPM) of *Zm*TrpA$^{SPM4\text{-}L6BX1}$ in the presence of the substrate IGP. Spheres represent identified positions that are conserved between *Zm*BX1/*Zm*TrpA$^{SPM4\text{-}L6BX1}$, whereas boxes represent different amino acids at conformationally relevant sites as identified by SPM. The position of the residue K168 (in the α-helix H5), is highlighted with a pink star. The substrate IGP is represented as sticks.

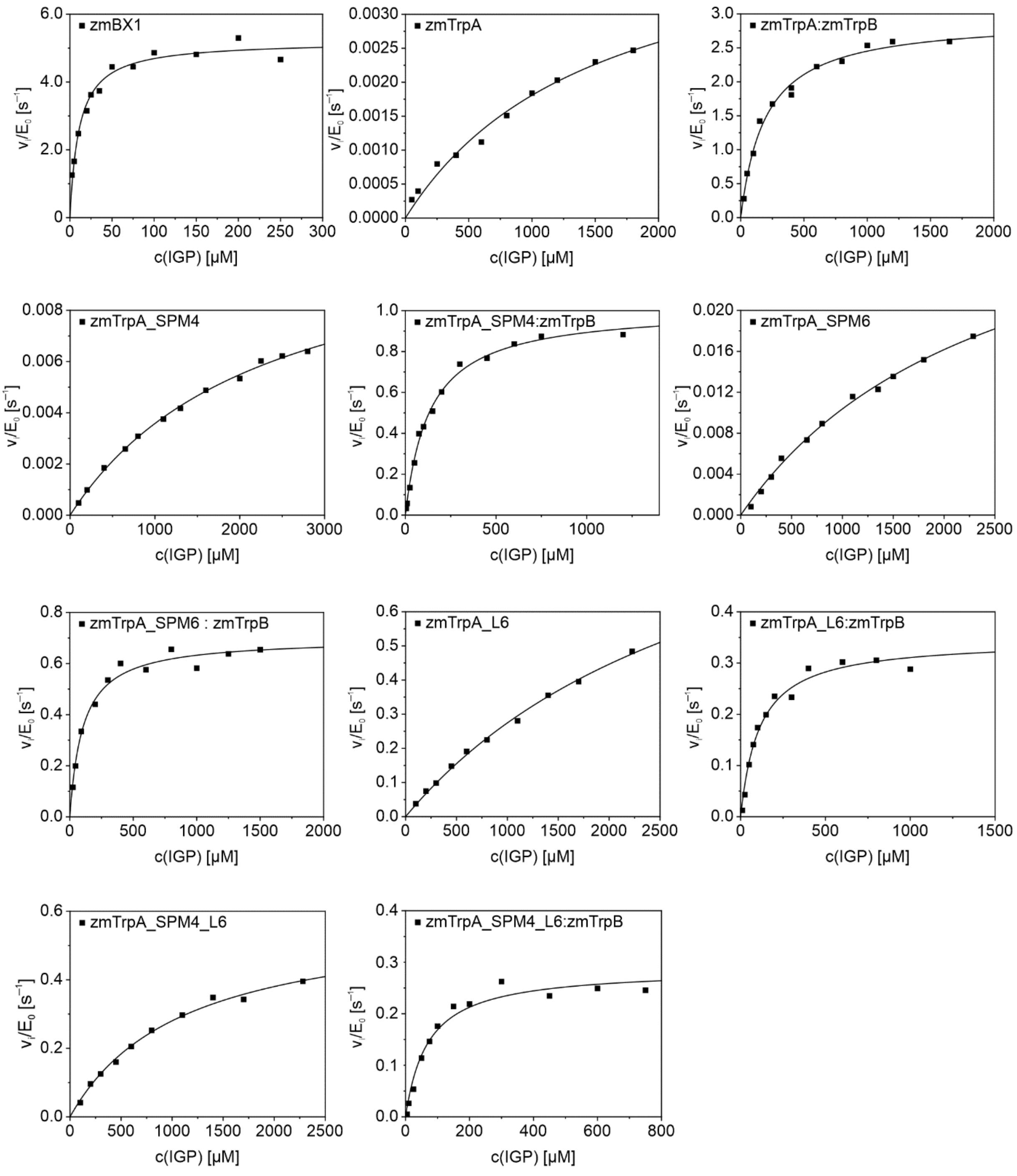

Figure S6. Michaelis-Menten curves that were used to determine the steady-state kinetic constants for *Zm*BX1, *Zm*TrpA and its variants in absence and presence of *Zm*TrpB. Experimental conditions are given in Table 1.

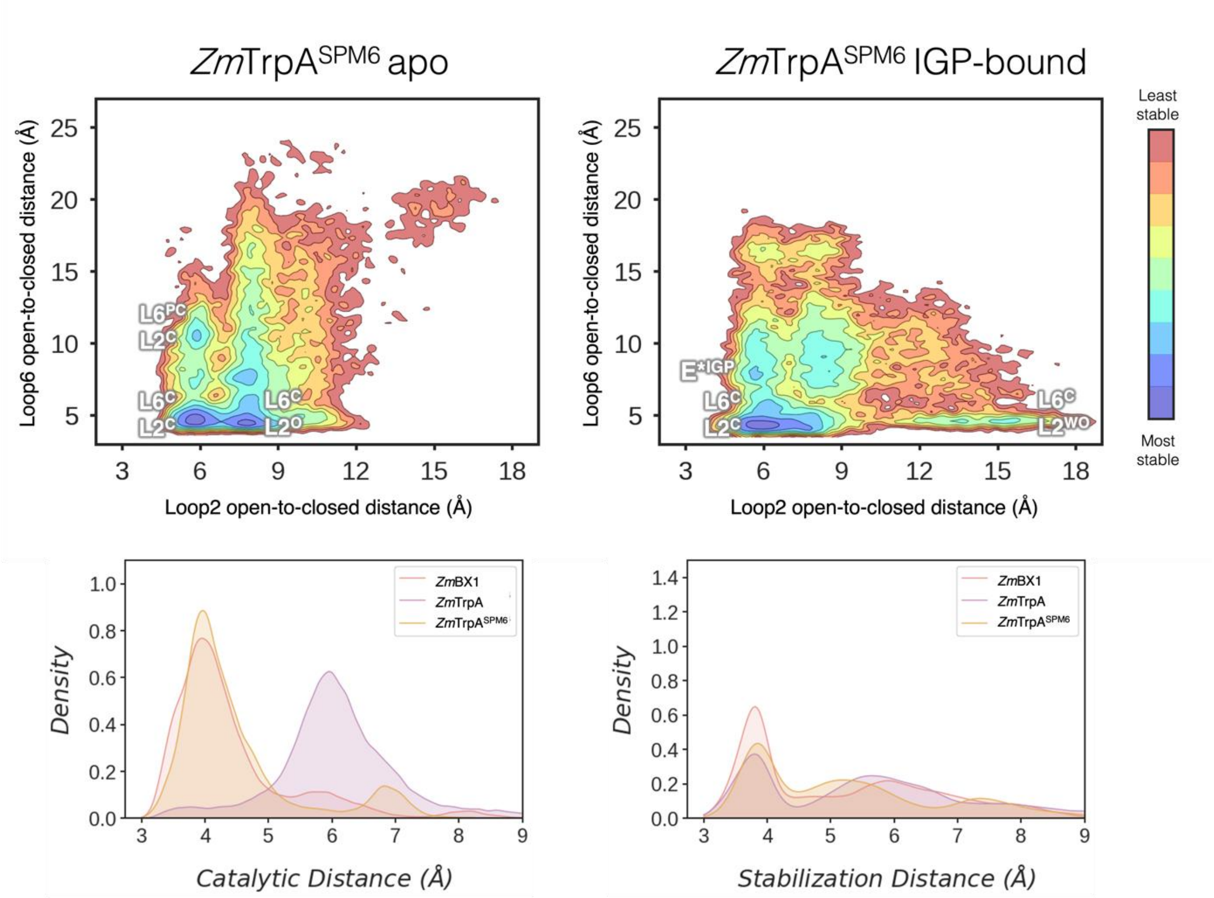

Figure S7. A. Reconstructed Free Energy Landscape (FEL) of *Zm*TrpA^{SPM6} in the apo (top left panel), and IGP-bound state (top right panel). For the FEL reconstruction, the distance between Thr183 and Gly62 residues describe the closed-to-open transition of L6 (y axis), and the distance between Leu59 and Asp130 for L2 opening (x axis) are used. Most stable conformations are colored in blue, whereas the least stable ones are depicted in red. Each minimum in the FEL is labelled according to the open (O)/closed (C) conformation of L6 and L2. The catalytically activated **E*^{IGP}** presenting both L6 and L2 in a closed conformation is labeled as **E*^{IGP}(L6^{C}L2^{C})**. Histogram of the catalytic distance between Glu50 and IGP (in Å, bottom left panel) and the catalytic stabilization distance between Asp60/61 and IGP (in Å, bottom right panel) for *Zm*BX1 (as reference, in pink), *Zm*TrpA (in purple), and *Zm*TrpA^{SPM6} (in orange).

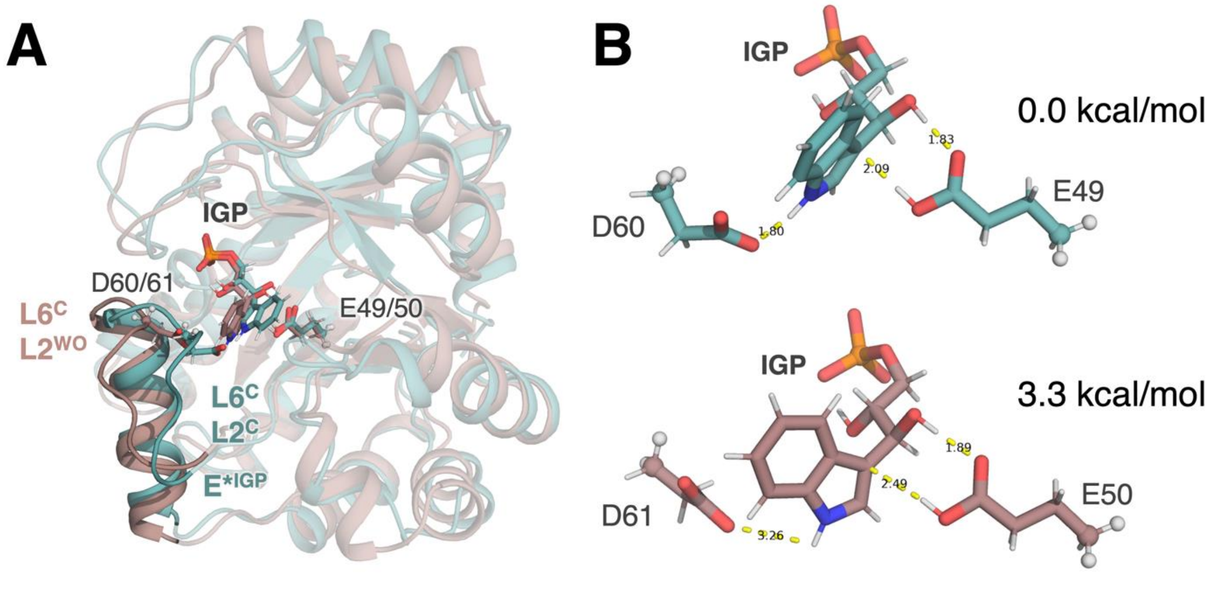

Figure S8. A. Overlay of the representative structures of the *Zm*BX1 minima: **L6^{C}L2^{C}** (**E*^{IGP}**) presenting L6 and L2 on a closed conformation (teal conformation); and **L6^{C}L2^{WO}** presenting L6 closed and L2 in a wide-open conformation (in brown). The residues considered for the DFT calculations are represented in sticks: Glh49/50, Asp60/61 and IGP. B. B3LYP-D3(BJ)/6-31G(d,p) optimized structures coming from the *Zm*BX1 minimum: **L6^{C}L2^{C}** (**E*^{IGP}**, top) and from the *Zm*TrpA minimum: **L6^{C}L2^{WO}** (bottom). Relative stabilities computed at the wB97XD/6-311+G(2d,2p)//B3LYP-D3(BJ)/6-31G(d,p) level of theory between both reactant complexes coming from the closed (teal) and open (brown) conformations are expressed in kcal/mol. Atoms maintained fixed along the optimizations are highlighted with spheres.

Table S1. FASTA amino acid sequences of the studied systems. Mutations are highlighted in orange.

*Zm*BX1	SRPVSDTMAALMAKGKTAFIPYITAGDPDLATTAEALRLLDGCGADVIEL<br>GVPCSDPYIDGPIIQASVARALASGTTMDAVLEMLREVTPELSCPVVLLS<br>YYKPIMSRSLAEMKEAGVHGLIVPDLPYVAAHSLWSEAKNNNLELVLLTT<br>PAIPEDRMKEITKASEGFVYLVSVNGVTGPRANVNPRVESLIQEVKKVTN<br>KPVAVGFGISKPEHVKQIAQWGADGVIIGSAMVRQLGEAASPKQGLRRL<br>EEYARGMKNALP

*Zm*TrpA	DKRSISGTFAELRQQGKTALIPFITAGDPDLATTAKALRILDACGSDVIEL<br>GVPYSDPLADGPVIQASATRALAKGTTFEDVISMVKGVIPDLSCPVALFT<br>YYNPILKRGVPNFMSIVKEAGVHGLVVPDVPLEETDVLRSEAAKNNLEL<br>VLLTTPTTPNERMEKIAQASEGFIYLVSTVGVTGTRANVSGKVQSLLQDI<br>KKVTEKPVAVGFGVSTPEHVRQIAGWGADGVIIGSAVMKTLEEAASPEE<br>GLKKLEQFAKNLKAALP
*Zm*TrpA^{L6BX1}	DKRSISGTFAELRQQGKTALIPFITAGDPDLATTAKALRILDACGSDVIELG<br>VPYSDPLADGPVIQASATRALAKGTTFEDVISMVKGVIPDLSCPVALFTY<br>YNPILKRGVPNFMSIVKEAGVHGLVVPDVPLEETDVLRSEAAKNNLELVL<br>LTTPTTPNERMEKIAQASEGFIYLVSVNGVTGPRANVNPRVQSLLQDIKK<br>VTEKPVAVGFGVSTPEHVRQIAGWGADGVIIGSAVMKTLEEAASPEEGL<br>KKLEQFAKNLKAALP
*Zm*TrpA^{SPM4}	DKRSISGTFAELRQQGKTALIPYITAGDPDLATTAKALRILDACGSDVIELG<br>VPYSDPLADGPVIQASATRALAKGTTFEDVISMVKGVIPDLSCPVALLSY<br>YNPILKRGVPNFMSIVKEAGVHGLVVPDVPLEETDVLRSEAAKNNLELVL<br>LTTPTTPNERMEKIAKASEGFIYLVSTVGVTGTRANVSGKVQSLLQDIKK<br>VTEKPVAVGFGVSTPEHVRQIAGWGADGVIIGSAVMKTLEEAASPEEGL<br>KKLEQFAKNLKAALP
*Zm*TrpA^{SPM6}	DKRSISGTFAELRQQGKTALIPYITAGDPDLATTAKALRILDACGSDVIELG<br>VPYSDPLADGPVIQASATRALAKGTTFEDVISMVKGVIPDLSCPVALLSY<br>YNPILKRGVPNFMSIVKEAGVHGLVVPDVPLEETDVLRSEAAKNNLELVL<br>LTTPTTPNERMEKIAKASEGFIYLVSVNGVTGTRANVSGKVQSLLQDIKK<br>VTEKPVAVGFGVSTPEHVRQIAGWGADGVIIGSAVMKTLEEAASPEEGL<br>KKLEQFAKNLKAALP
*Zm*TrpA$^{SPM4-L6BX1}$	DKRSISGTFAELRQQGKTALIPYITAGDPDLATTAKALRILDACGSDVIELG<br>VPYSDPLADGPVIQASATRALAKGTTFEDVISMVKGVIPDLSCPVALLSY<br>YNPILKRGVPNFMSIVKEAGVHGLVVPDVPLEETDVLRSEAAKNNLELVL<br>LTTPTTPNERMEKIAKASEGFIYLVSVNGVTGPRANVNPRVQSLLQDIKK<br>VTEKPVAVGFGVSTPEHVRQIAGWGADGVIIGSAVMKTLEEAASPEEGL<br>KKLEQFAKNLKAALP

References

(1) Jumper, J.; Evans, R.; Pritzel, A.; Green, T.; Figurnov, M.; Ronneberger, O.; Tunyasuvunakool, K.; Bates, R.; Žídek, A.; Potapenko, A.; Bridgland, A.; Meyer, C.; Kohl, S. A. A.; Ballard, A. J.; Cowie, A.; Romera-Paredes, B.; Nikolov, S.; Jain, R.; Adler, J.; Back, T.; Petersen, S.; Reiman, D.; Clancy, E.; Zielinski, M.; Steinegger, M.; Pacholska, M.; Berghammer, T.; Bodenstein, S.; Silver, D.; Vinyals, O.; Senior, A. W.; Kavukcuoglu, K.; Kohli, P.; Hassabis, D. Highly accurate protein structure prediction with AlphaFold. *Nature* **2021**, *596*, 583-589.

(2) Ester, M.; Kriegel, H.-P.; Sander, J.; Xu, X. A Density-Based Algorithm for Discovering Clusters in Large Spatial Databases with Noise. In Proc. of 2nd International Conference on Knowledge Discovery and, 1996.

(3) Jukič, M.; Konc, J.; Gobec, S.; Janežič, D. Identification of Conserved Water Sites in Protein Structures for Drug Design. *Journal of Chemical Information and Modeling* **2017**, *57*, 3094-3103.

(4) Pedregosa, F.; Varoquaux, G.; Gramfort, A.; Michel, V.; Thirion, B.; Grisel, O.; Blondel, M.; Prettenhofer, P.; Weiss, R.; Dubourg, V.; Vanderplas, J.; Passos, A.; Cournapeau, D.; Brucher, M.; Perrot, M.; Duchesnay, E. Scikit-learn: Machine Learning in {P}ython. *J. Mach. Learn. Res.* **2011**, *12*, 2825-2830.

(5) *AMBER 2020*; University of California, San Francisco, 2020. (accessed.

(6) Wang, J.; Wolf, R. M.; Caldwell, J. W.; Kollman, P. A.; Case, D. A. Development and testing of a general amber force field. *Journal of Computational Chemistry* **2004**, *25*, 1157-1174.

(7) Schutz, C. N.; Warshel, A. What are the dielectric “constants” of proteins and how to validate electrostatic models? *Proteins* **2001**, *44*, 400-417.

(8) Bayly, C. I.; Cieplak, P.; Cornell, W.; Kollman, P. A. A well-behaved electrostatic potential based method using charge restraints for deriving atomic charges: the RESP model. *The Journal of Physical Chemistry* **1993**, *97*, 10269-10280.

(9) Singh, U. C.; Kollman, P. A. An approach to computing electrostatic charges for molecules. *Journal of Computational Chemistry* **1984**, *5*, 129-145.

(10) *Gaussian 16 Rev. C.01*; Wallingford, CT, 2016. (accessed.

(11) Olsson, M. H. M.; Søndergaard, C. R.; Rostkowski, M.; Jensen, J. H. PROPKA3: Consistent Treatment of Internal and Surface Residues in Empirical pKa Predictions. *Journal of Chemical Theory and Computation* **2011**, *7*, 525-537.

(12) Søndergaard, C. R.; Olsson, M. H. M.; Rostkowski, M.; Jensen, J. H. Improved Treatment of Ligands and Coupling Effects in Empirical Calculation and Rationalization of pKa Values. *Journal of Chemical Theory and Computation* **2011**, *7*, 2284-2295.

(13) Dunn, M. F. Allosteric regulation of substrate channeling and catalysis in the tryptophan synthase bienzyme complex. *Arch. Biochem. Biophys.* **2012**, *519*, 154-166.

(14) Tian, C.; Kasavajhala, K.; Belfon, K. A. A.; Raguette, L.; Huang, H.; Migues, A. N.; Bickel, J.; Wang, Y.; Pincay, J.; Wu, Q.; Simmerling, C. ff19SB: Amino-Acid-Specific Protein Backbone Parameters Trained against Quantum Mechanics Energy Surfaces in Solution. *Journal of Chemical Theory and Computation* **2020**, *16*, 528-552.

(15) Roe, D. R.; Cheatham, T. E. PTRAJ and CPPTRAJ: Software for Processing and Analysis of Molecular Dynamics Trajectory Data. *Journal of Chemical Theory and Computation* **2013**, *9*, 3084-3095.

(16) Darden, T.; York, D.; Pedersen, L. Particle mesh Ewald: An N·log(N) method for Ewald sums in large systems. *The Journal of Chemical Physics* **1993**, *98*, 10089-10092.

(17) McGibbon, Robert T.; Beauchamp, Kyle A.; Harrigan, Matthew P.; Klein, C.; Swails, Jason M.; Hernández, Carlos X.; Schwantes, Christian R.; Wang, L.-P.; Lane, Thomas J.;

Pande, Vijay S. MDTraj: A Modern Open Library for the Analysis of Molecular Dynamics Trajectories. *Biophys J* **2015**, *109*, 1528-1532.
(18) Nguyen, H.; Roe, D. R.; Swails, J.; Case, D. A. PYTRAJ v1.0.0.dev1: Interactive data analysis for molecular dynamics simulations (v1.0.0.dev1). *Zenodo* **2016**.
(19) Gowers, R. J.; Linke, M.; Barnoud, J.; Reddy, T. J. E.; Melo, M. N.; Seyler, S. L.; Domanski, J.; Dotson, D. L.; Buchouz, S.; Kenney, I. M.; Beckstein, O. MDAnalysis: a Python package for the rapid analysis of molecular dynamics simulations. *Proc. of the 15th python in science conf.* **2016**, 98-105.
(20) Scherer, M. K.; Trendelkamp-Schroer, B.; Paul, F.; Pérez-Hernández, G.; Hoffmann, M.; Plattner, N.; Wehmeyer, C.; Prinz, J.-H.; Noé, F. PyEMMA 2: A Software Package for Estimation, Validation, and Analysis of Markov Models. *Journal of Chemical Theory and Computation* **2015**, *11*, 5525-5542.
(21) Osuna, S. The challenge of predicting distal active site mutations in computational enzyme design. *Wiley Interdiscip. Rev. Comput. Mol. Sci.* **2021**, e1502.
(22) Romero-Rivera, A.; Garcia-Borràs, M.; Osuna, S. Role of Conformational Dynamics in the Evolution of Retro-Aldolase Activity. *ACS Catal.* **2017**, *7*, 8524-8532.
(23) Grimme, S.; Ehrlich, S.; Goerigk, L. Effect of the damping function in dispersion corrected density functional theory. *Journal of Computational Chemistry* **2011**, *32*, 1456-1465.
(24) Becke, A. D. Density-functional thermochemistry. III. The role of exact exchange. *The Journal of Chemical Physics* **1993**, *98*, 5648-5652.
(25) Lee, C.; Yang, W.; Parr, R. G. Development of the Colle-Salvetti correlation-energy formula into a functional of the electron density. *Physical Review B* **1988**, *37*, 785-789.
(26) Chai, J.-D.; Head-Gordon, M. Long-range corrected hybrid density functionals with damped atom–atom dispersion corrections. *Physical Chemistry Chemical Physics* **2008**, *10*, 6615-6620, 10.1039/B810189B.
(27) Rohweder, B.; Semmelmann, F.; Endres, C.; Sterner, R. Standardized cloning vectors for protein production and generation of large gene libraries in Escherichia coli. *BioTechniques* **2018**, *64*, 24-26.
(28) Schupfner, M.; Busch, F.; Wysocki, V. H.; Sterner, R. Generation of a Stand-Alone Tryptophan Synthase α-Subunit by Mimicking an Evolutionary Blueprint. *ChemBioChem* **2019**, *20*, 2747-2751.
(29) Creighton, T. E. A Steady-State Kinetic Investigation of the Reaction Mechanism of the Tryptophan Synthetase of Escherichia coli. *European Journal of Biochemistry* **1970**, *13*, 1-10.
